# Positive and Negative Regulatory Roles of C-Terminal Src Kinase (CSK) in FcεRI-Mediated Mast Cell Activation, Independent of the Transmembrane Adaptor PAG/CSK-Binding Protein

**DOI:** 10.3389/fimmu.2018.01771

**Published:** 2018-08-02

**Authors:** Lucie Potuckova, Lubica Draberova, Ivana Halova, Tomas Paulenda, Petr Draber

**Affiliations:** Department of Signal Transduction, Institute of Molecular Genetics of the Czech Academy of Sciences, Prague, Czechia

**Keywords:** mast cell, degranulation, cytokines, C-terminal Src kinase, phosphoprotein associated with glycosphingolipid-enriched microdomains, LYN, SHP-1, STAT5

## Abstract

C-terminal Src kinase (CSK) is a major negative regulator of Src family tyrosine kinases (SFKs) that play critical roles in immunoreceptor signaling. CSK is brought in contiguity to the plasma membrane-bound SFKs *via* binding to transmembrane adaptor PAG, also known as CSK-binding protein. The recent finding that PAG can function as a positive regulator of the high-affinity IgE receptor (FcεRI)-mediated mast cell signaling suggested that PAG and CSK have some non-overlapping regulatory functions in mast cell activation. To determine the regulatory roles of CSK in FcεRI signaling, we derived bone marrow-derived mast cells (BMMCs) with reduced or enhanced expression of CSK from wild-type (WT) or PAG knockout (KO) mice and analyzed their FcεRI-mediated activation events. We found that in contrast to PAG-KO cells, antigen-activated BMMCs with CSK knockdown (KD) exhibited significantly higher degranulation, calcium response, and tyrosine phosphorylation of FcεRI, SYK, and phospholipase C. Interestingly, FcεRI-mediated events in BMMCs with PAG-KO were restored upon CSK silencing. BMMCs with CSK-KD/PAG-KO resembled BMMCs with CSK-KD alone. Unexpectedly, cells with CSK-KD showed reduced kinase activity of LYN and decreased phosphorylation of transcription factor STAT5. This was accompanied by impaired production of proinflammatory cytokines and chemokines in antigen-activated cells. In line with this, BMMCs with CSK-KD exhibited enhanced phosphorylation of protein phosphatase SHP-1, which provides a negative feedback loop for regulating phosphorylation of STAT5 and LYN kinase activity. Furthermore, we found that in WT BMMCs SHP-1 forms complexes containing LYN, CSK, and STAT5. Altogether, our data demonstrate that in FcεRI-activated mast cells CSK is a negative regulator of degranulation and chemotaxis, but a positive regulator of adhesion to fibronectin and production of proinflammatory cytokines. Some of these pathways are not dependent on the presence of PAG.

## Introduction

The aggregation of high-affinity IgE receptor (FcɛRI)–IgE complexes by multivalent antigen in mast cells leads to the release of a variety of mediators that play important roles in innate and adaptive immunity (1). Activated mast cells release mediators pre-stored in cytoplasmic granules, such as histamine, proteases, proteoglycans, and cytokines, as well as *de novo* synthesized lipids, cytokines, and chemokines. The first biochemically well-defined step in FcɛRI-mediated cell activation is tyrosine phosphorylation of immunoreceptor tyrosine-based activation motifs (ITAMs) in the cytoplasmic domains of FcɛRI β and γ subunits by Src family kinase (SFK) LYN, followed by recruitment of protein tyrosine kinase (PTK) SYK to FcεRI γ and its activation. LYN and SYK, together with FYN and some other PTKs, phosphorylate the tyrosine motifs of transmembrane adaptor proteins (TRAP) such as linker for activation of T cells [LAT; official name LAT1 (2)], non-T cell activation linker [NTAL; official name LAT2 (3)], and phosphoprotein associated with glycosphingolipid-enriched microdomains [PAG; official name PHAG1, also known as C-terminal Src kinase (CSK)-binding protein (CBP) (4–6)], which serve as anchors for other signal-transduction molecules that govern the biochemical signals responsible for initiating cell degranulation and cytokine and chemokine production. How exactly phosphorylation of the FcεRI subunits by LYN kinase is initiated is not completely understood and several models have been proposed, including the transphosphorylation model (7), lipid raft model (8), and PTK-protein tyrosine phosphatase (PTP) interplay model (9, 10).

The catalytic activity of LYN kinase is tightly regulated by phosphorylation/dephosphorylation of two conserved tyrosines at positions 397 (Y^397^) and 508 (Y^508^) (11–13). Phosphorylation of Y^397^ stabilizes the activation loop of the catalytic domain and increases the LYN activity. In contrast, phosphorylation of Y^508^ at the C-terminus of LYN promotes a structure in which intramolecular binding of the Src homology 2 (SH2) domain with phosphorylated Y^508^ stabilizes the inactive conformation of the catalytic domain. Y^397^ of LYN is autophosphorylated *in trans*, whereas Y^508^ phosphorylation is catalyzed by the CSK. The critical role of CSK in cell physiology was demonstrated by the phenotype of mice lacking CSK. These mice die in early embryonic stages and their tissues possess Src family tyrosine kinases (SFKs) with increased enzymatic activity (14–16). When CSK was inactivated in immature thymocytes, T cell development was significantly impaired (17). Furthermore, mice possessing granulocytes with CSK inactivated by conditional mutagenesis developed acute inflammatory responses (18).

In contrast to SFKs, which are anchored to the plasma membrane *via* their N-terminal myristoyl and/or palmitate moieties, CSK lacks the transmembrane domain and fatty acyl modifications and is predominantly localized in the cytosol (16). Thus, recruitment of cytosolic CSK to the vicinity of the plasma membrane-bound SFKs is involved in CSK-SFK cross-talk. An important anchor of CSK in the plasma membrane and regulator of CSK function is PAG (4). PAG is expressed ubiquitously, and like other TRAPs, has a short extracellular domain, a transmembrane domain followed by a palmitoylation site, and a cytoplasmic domain with 10 potential tyrosine phosphorylation sites. In mouse PAG, phosphorylated Y^314^ binds with CSK through its SH2 domain. Based on initial studies it has been postulated that PAG in resting T cells is constitutively phosphorylated and associated with CSK, which phosphorylates C-terminal tyrosines of SFKs in the vicinity of PAG and in this way inactivates them. Activation through the T cell receptor (TCR) leads to rapid PAG dephosphorylation, which results in the release of CSK from PAG and relief of CSK-mediated inhibition of SFKs (19).

In contrast to T cells, in mast cells FcεRI activation results in rapid tyrosine phosphorylation of PAG, peaking at 2–3 min after triggering, and then returns to the basal levels. This suggests that together with LYN and CSK, PAG initiates a negative regulatory loop contributing to the signal termination (20, 21). It should be noted, however, that recent experiments have shown that even in resting T cells, PAG displays a low basal level of tyrosine phosphorylation that increases upon T cell activation and that the inhibitory function of PAG on TCR is seen in effector but not in naive T cells (22, 23).

Our recent study showed that bone marrow-derived mast cells (BMMCs) with PAG knockout (KO) or PAG knockdown (KD) exhibited impaired antigen-induced degranulation, calcium response, tyrosine phosphorylation of FcεRI, SYK, and some other signal-transduction molecules, production of several cytokines and chemokines, and chemotaxis. At the same time, the enzymatic activities of LYN and FYN kinases were increased in nonactivated cells, suggesting involvement of a LYN and/or FYN-dependent negative regulatory loop (24). These data, together with our finding of enhanced degranulation and c-KIT receptor phosphorylation in stem cell factor (SCF)-activated BMMCs with PAG-KO, suggested that PAG can function as a positive or negative regulator of mast cell signaling, depending on the signaling pathway involved.

To better understand the contribution of CSK and PAG in FcεRI-mediated signaling, in this study we examined the antigen-induced activation events in BMMCs from WT and PAG-KO mice with reduced or enhanced expression of CSK and corresponding controls. We also analyzed the involvement of CSK and PAG in adhesion to fibronectin and chemotaxis toward antigen and SCF. Our data indicate that CSK and PAG in mast cells are, respectively, negative and positive regulators of events leading to degranulation and chemotaxis toward antigen and SCF, whereas they are both positive regulators of FcɛRI-induced production of cytokines and chemokines. The combined data indicate that CSK regulates production of proinflammatory cytokines and chemokines through the LYN/SHP-1/STAT5 axis.

## Materials and Methods

### Antibodies and Reagents

The following monoclonal antibodies (mAbs) were used: mouse IgE mAb specific for 2,4,6-trinitrophenol (TNP) clone IGEL b4.1 (25), anti-FcεRI β chain JRK (26), anti-LYN (27), and SYK-specific mAb (28). Polyclonal antibodies specific for NTAL, LAT, and LYN were prepared in our laboratory by immunization of rabbits with the corresponding recombinant proteins or their fragments (29). Rabbit anti-IgE was prepared by immunization with whole IGEL b4.1. Polyclonal antibodies specific for CSK [catalog number (Cat. No.) sc-286], STAT5 (Cat. No. sc-835), SHP-1 (Cat. No. sc-287), FYN (Cat. No. sc-16), phospholipase C (PLC) γ1 (Cat. No. sc-81), phospho-PLC γ1^Y783^ (Cat. No. sc-12943), and GRB2 (Cat. No. sc-255), as well as horseradish peroxidase (HRP)-conjugated goat anti-mouse IgG (Cat. No. sc-2005) and goat anti-rabbit IgG (Cat. No. sc-2004) were obtained from Santa Cruz Biotechnology Inc. Antibodies specific for phospho-Lyn^Y507^ (in mouse Lyn^Y508^; Cat. No. 2731), phospho-SFKs^Y416^ (in mouse LYN^Y397^; Cat. No. 2101), phospho-SYK^Y525/Y526^ (Cat. No. 2711), phospho-STAT5^Y694^ (Cat. No. 9351), and phospho-SHP-1^Y564^ (Cat. No. 8849) were obtained from Cell Signaling. Phospho-LAT^Y191^ (Cat. No. 07-278) and Protein G HRP conjugate (Cat. No. 18-161) were obtained from Millipore. Anti-mouse β1-integrin-specific antibody HM β1-1 (Cat. No. 553837) was purchased from BD Biosciences. Alexa Fluor (AF)488-conjugated goat Ab specific for hamster IgG (Cat. No. A-21110) or mouse IgG (Cat. No. A-11001) were obtained from Life Technologies. HRP-conjugated anti-phosphotyrosine mAb (PY20; Cat. No. 610012) and V450-conjugated rat anti-mouse CD107a (Cat. No. 560648) were obtained from BD Biosciences. Anti-mouse FcεRI-fluorescein isothiocyanate (FITC; Cat. No. 11-5898) and anti-mouse c-KIT (CD117)-allophycocyanin (APC; Cat. No. 17-1171) conjugates were obtained from eBiosciences. Polyclonal antibodies specific for mouse tumor necrosis factor (TNF)-α (Cat. No. 500-P64), interleukin (IL)-13 (Cat. No. 500-P178), recombinant mouse TNF-α (Cat. No. 315-01A), recombinant IL-13 (Cat. No. 210-13), and recombinant IL-6 (Cat. No. 216-16) were obtained from PeproTech. Murine anti-IL-6 (Cat. No. 554400) was purchased from Becton Dickinson. Colloidal gold nanoparticles (Au-NPs; diameter, 30 nm; Cat. No. EM.GC30), consisting of approximately 2 × 10^11^ Au-NPs/ml, were obtained from BB International. TNP-bovine serum albumin (BSA) conjugate (15–25 mol TNP/mol BSA) was produced as described previously (30). ^45^Ca (specific activity, 773 MBq/mg Ca^2+^) and [γ-^32^P] ATP (specific activity, 222 TBq/mmol) were purchased from the Institute of Isotopes Co., Ltd. (Budapest, Hungary). All other reagents were from Sigma-Aldrich.

### Mice and Cells

Mice deficient in PAG and their WT littermates of C57BL/6 genotype were used in this study. PAG-KO mice were generated by the modified bacterial artificial chromosome technology as previously described (24, 31, 32). Bone marrow cells were isolated from the femurs and tibias of 8–12-week-old mice. The cells were cultured for 8–12 weeks in RPMI-1640 medium supplemented with 100 U/ml penicillin, 100 µg/ml streptomycin, 71 µM 2-mercaptoethanol, minimum essential medium (MEM) non-essential amino acids, 0.7 mM sodium pyruvate, 2.5 mM l-glutamine, 12 mM d-glucose, recombinant mouse SCF (15 ng/ml, PeproTech EC), mouse recombinant IL-3 (15 ng/ml, PeproTech EC), and 10% fetal calf serum (FCS). Stable mast cell lines derived from mouse WT BMMCs (Lyn^+/+^) or mouse LYN-KO BMMCs (Lyn^−/−^), were obtained from Hibbs et al. (11) and were used for experiments with CSK-OE if not stated otherwise. These cell lines were cultured in RPMI-1640 medium supplemented as described above, except that SCF was omitted.

### Lentiviral Vectors and Gene Transduction

Lentiviral transductions were done as described previously using HEK 293 T/17 packaging cells for virus preparation (24). A set of murine CSK-specific short hairpin (sh) RNAs constructs based on pLKO.1 vector [TRCN0000023735 (shRNA 35), TRCN0000023736 (shRNA 36), TRCN0000361164 (shRNA 64), and TRCN0000321790 (shRNA 90)] were purchased from Open Biosystems. Cells were transduced with individual shRNAs or with a pool of shRNAs prepared by mixing shRNAs 35, 36, 64, and 90 in equimolar ratios. Individual constructs or the pool of the constructs (14 µg) was mixed with OptiMEM (1 ml; Invitrogen), 21 µl of lentiviral packaging mix (Invitrogen), and 105 µl polyethyleneimine (Sigma) and virus was produced and transduced as described previously (24). The pilot degranulation experiments were performed with individual shRNA vectors, but because the individual vectors gave similar results as the pooled shRNA vectors, further experiments were done with the shRNA pool denoted in further text as CSK-KD. For experiments with stable CSK overexpression, a pMX plasmid encoding human Csk (hCSK) was used for recloning the hCSK-mCherry cassette into the vector pCDH-CMV-MCS-EF1-Puro (Systembio; pCDH). For PCR, the forward primer 5′-AAATCTAGAGCCACCATGTCAGCAATACAGGCC-3′ (the XbaI restriction site is underlined) and the reverse primer 3′-TTTGCGGCCGCCTACTTGTACAGCTCGTCCAT-5′ (the NotI restriction site is underlined) were used. The construct was verified by sequencing and denoted hCSK-mCherry. In all experiments, empty pLKO.1 and/or non-target (NTG) vectors or empty pCDH vectors were used as controls. In some experiments related to CD107a and β-glucuronidase detection, we also used the pLKO.1 vector containing NTG shRNA (Sigma-Aldrich) as a control.

### Flow Cytometry Analysis

To quantify the surface presence of FcɛRI and c-KIT, BMMCs were double-stained with anti-mouse FcɛRI-FITC and anti-mouse c-KIT-allophycocyanin antibodies for 30 min on ice. After labeling, the cells were washed three times with ice-cold phosphate-buffered saline (PBS) and analyzed with an LSRII flow cytometer (BD Biosciences) and further processed using FlowJo software (Ashland, OR, USA). For analysis of the CD107a presence on the cell surface, BMMCs were activated or not with antigen (10–100 ng/ml) for 10 min at 37°C. Activation was stopped by centrifugation at 4°C. The cells were then resuspended in 50 µl of PBS-1% BSA containing 200-fold-diluted V450-conjugated rat anti-mouse CD107a and stained on ice for 30 min. After washing with PBS, the cells were examined as described above. For analysis of IgE internalization, IgE-sensitized cells were activated with antigen (500 ng/ml) or not for various time intervals (5, 15, or 30 min) at 37°C. Activation was stopped by centrifugation at 4°C (300 × *g*; 3 min), cells were fixed with 4% paraformaldehyde for 15 min and washed with PBS. IgE was visualized using AF488-conjugated anti-mouse IgG (H + L) antibody, which cross-reacts with IgE, as previously describe (33). To quantify the surface abundance of β1-integrin, the cells were exposed for 30 min on ice to β1-integrin-specific antibodies HM β1-1 and incubated for 30 min with AF488-conjugated goat Ab specific for hamster IgG. After the incubation, the cells were washed in ice-cold PBS and evaluated as previously described (34).

### β-Glucuronidase Release and Ca^2+^ Response

Bone marrow-derived mast cells were cultured for 4 h in SCF- and IL-3-free medium supplemented with TNP-specific IgE (1 µg/ml). The IgE-sensitized cells were washed, and degranulation assay, based on evaluation of β-glucuronidase release, was performed and evaluated as previously described (24). Fluorescence was determined by an Infinite 200M (Tecan) plate reader at 355-nm excitation and 460-nm emission wavelengths. Calcium response was analyzed using Fura-2, AM (Life Technologies) cytoplasmic reporter, as described previously (24). The levels of intracellular Ca^2+^ were determined by spectrofluorometry using the Infinite 200M plate reader with excitation wavelengths at 340 and 380 nm and with constant emission at 510 nm.

### Cytokine and Chemokine Detection

mRNA was extracted from IgE-sensitized and antigen-activated (100 ng/ml) BMMCs using a TurboCapture 96 mRNA kit or RNeasy miniKit (Qiagen). Single-stranded cDNA was synthesized with M-MLV reverse transcriptase (Invitrogen) according to the manufacturer’s instructions. Real-time quantitative polymerase chain reaction (RT-qPCR) amplifications of cDNAs and oligonucleotide primers used were described previously (24). Actin, GAPDH, and ubiquitin were used as reference genes, and the expression levels of TNF-α, IL-13, IL-6, CCL3, and CCL4 mRNAs were normalized to the geometric mean of the reference genes in nonactivated control cells. For quantification of cytokines secreted into the media, an immuno-PCR method with gold nanoparticles armed with immobilized DNA template and cytokine-specific antibodies were used as described previously (35), with some modifications. Briefly, anti-TNF-α, anti-IL-6, or anti-IL-13 in 100 mM borate buffer (pH 9.5) was dispensed in 50-µl aliquots into the wells of a real-time 96-well plate (Eppendorf). After overnight incubation at 4°C, each well was washed four times with 200 µl of Tris-buffered saline (TBS; 10 mM Tris–HCl, pH 7.4, 150 mM NaCl) containing 0.05% Tween 20 (TBST), and the remaining binding sites were blocked by 2-h incubation at 37°C with TBST supplemented with 2% BSA. After washing, 50 µl of serial dilutions (0.1–100 ng/ml) of recombinant TNF-α, IL-6, or IL-13 (used for construction of calibration curves) or the tested samples diluted in PBS-1% BSA was added. The samples were incubated for 1 h at 37°C, and after washing with TBST, 50 µl of Au-NPs armed with thiolated DNA oligonucleotide template and with the corresponding cytokine-specific antibody was applied into each well. The wells were incubated for 1 h at 37°C and washed with TBST and deionized water. Fifty-µl aliquots of qPCR master mix solution (see above) supplemented with 60 nM of the corresponding oligonucleotide primers were then dispensed into each well, as described previously (24). The plates were sealed, and the amount of template DNA bound to antigen-anchored functionalized Au-NPs was evaluated by real-time PCR using a Realplex4 Mastercycler apparatus (Eppendorf). For calculation of cytokine concentrations, the corresponding cycle threshold values of the cytokines were substituted into the regression equations obtained from the calibration curves constructed from the concentration series of appropriate recombinant proteins.

### Cell Chemotaxis and Adhesion Assay

Chemotaxis responses were assayed in 24-well transwell chamber (Corning) with 8 µm-pore-size polycarbonate filters in the upper wells. Chemoattractants, antigen (250 ng/ml) or SCF (50 ng/ml), were added to the lower compartments, and cell migration was assessed as previously described (24). Adhesion to fibronectin was quantified in 96-well plate coated overnight with fibronectin (10 µg/ml in PBS), blocked with 4% BSA in PBS (1 h at 37°C), and washed twice with PBS. IgE-sensitized BMMCs were loaded with Calcein-AM (4 μM) for 30 min at 37°C, washed and transferred into the fibronectin-coated wells (10^5^ cells/well). After activation with antigen (10–100 ng/ml, 30 min) or SCF (50 and 100 ng/ml, 30 min), unbound cells were washed out using a microplate washer (HydroSpeed, TECAN) and the bound cells were determined using an Infinite 200M Fluorometer with excitation and emission filters at 485 and 538 nm, respectively.

### Immunoprecipitation and Immunoblotting

Whole-cell extracts from nonactivated or antigen (250 ng/ml) activated cells were prepared by solubilizing cell pellets in sodium dodecyl sulfate (SDS) sample buffer, followed by sonication and boiling of samples for 5 min. Proteins were size-fractionated by 10% SDS-polyacrylamide gel electrophoresis, electrophoretically transferred onto nitrocellulose membrane, and analyzed by immunoblotting with protein- or phosphoprotein-specific antibodies, followed by HRP-conjugated anti-mouse or anti-rabbit IgG antibodies. Phosphorylation levels were normalized to the corresponding loading proteins. In immunoprecipitation experiments, cells were solubilized in ice-cold immunoprecipitation buffer (25 mM Tris–HCl, pH 8.0, 140 mM NaCl, 1 mM Na_3_VO_4_, 2 mM EDTA, 1 µg/ml aprotinin, 1 µg/ml leupeptin, and 1 mM phenylmethylsulfonyl fluoride) supplemented with 0.2% Triton X-100 (for FcεRI immunoprecipitation), 1% n-dodecyl-β-d-maltoside and 1% Nonidet P-40 (for LYN and FYN immmunoprecipitations), or 0.5% Triton X-100 (for coimmunoprecipitation experiments in which the buffer contained instead of Tris–HCl, pH 8.0, Tris–HCl, pH 7.5). After incubation on ice for 30 min, the lysates were centrifuged (16,000 × *g* for 5 min at 4°C) and postnuclear supernatants were immunoprecipitated with the corresponding antibodies prebound to UltraLink-immobilized protein A (Pierce, Thermo Scientific). The immunoprecipitated proteins were size-fractionated by SDS-PAGE as described above, transferred to nitrocellulose membrane and immunoblotted with PY20-HRP or with protein-specific antibodies, followed by HRP-conjugated anti-mouse or anti-rabbit IgG antibody. The HRP signal was detected by a Luminescent Image Analyzer LAS 3000 (Fuji Photo Film Co.). Aida software (Raytest GmbH) was used for signal quantification. The amount of phosphorylated proteins was normalized to the amount of immunoprecipitated proteins after stripping of the membranes, followed by development with the corresponding antibodies.

### Immunocomplex Kinase Assay

The *in vitro* kinase assays were performed as previously described (24, 36), with some modifications. FcεRI, LYN, and FYN were solubilized in immunoprecipitation buffer-1% Brij 96. Proteins immobilized on antibody-armed protein A beads were washed with kinase buffer (25 mM HEPES-NaOH, pH 7.2, 3 mM MnCl_2_, 0.1% Nonidet P-40, 100 mM Na_3_VO_4_, and 20 mM MgCl_2_) and then resuspended in 25 µl kinase buffer supplemented with 2.5 μCi (92.5 kBq) of [γ-^32^P]ATP, 100 µM ATP, and 0.5 µg/µl of acid-denatured enolase as the exogenous substrate. Immunoprecipitates were eluted with reducing SDS-PAGE sample buffer. The ^32^P-labeled proteins were size-fractionated by SDS-PAGE, transferred to nitrocellulose membrane, and visualized by autoradiography. Films were quantified with Aida image analyzer software (Raytest).

### Statistical Analyses

Results are expressed as means ± SEM. Statistical significance was evaluated by unpaired two-tailed Student’s *t*-test for comparison between two groups. In experiments where more than two groups were compared, the significance of intergroup differences was determined by one-way ANOVA with Tukey’s post-test. Comparison of changes over time between different groups was performed using two-way ANOVA with Bonferroni post-test. *P* values of less than 0.05 were considered significant. GraphPad Prism 5 (GraphPad Software, La Jolla, CA, USA) or Microsoft Excel 2010 were used for statistical analyses. Statistical method used and number of replicates are indicated in the corresponding figure legends.

## Results

### Production of BMMCs With Reduced or Enhanced Expression of CSK

To determine the role of CSK in mast cell signaling, we prepared BMMCs from C57BL/6 mice with reduced or enhanced expression of CSK. For CSK silencing we used four CSK-specific shRNAs (35, 36, 64, and 90) in lentiviral pLKO.1 vector. As controls, BMMCs transduced with empty pLKO.1 vector or pLKO.1 vector containing an NTG sequence were used. Because there were no significant differences between the results obtained with these different control vectors, the data were pooled and are presented together as control pLKO.1 vector. Lentiviral-mediated delivery of individual CSK-specific shRNAs resulted in reduction of CSK protein to 26 ± 4% (shRNA 35; mean ± SEM), 18 ± 6% (shRNA 36), 26 ± 5% (shRNA 64), and 36 ± 4% (shRNA 90) when compared to cells transduced with control pLKO.1 (Figures S1A,B in Supplementary Material). BMMCs were also transduced with a pool of all four CSK-specific shRNAs, which reduced the level of CSK in these cells on average to 19 ± 3% of controls (Figures S1C,D in Supplementary Material). To get enhanced expression of CSK, BMMCs were transduced with pCDH lentiviral vector containing human Csk cDNA tagged with mCherry. Empty pCDH vector was used as a control. Ten days after transduction and growing in media with puromycin, more than 90% of cells transduced with the hCSK-mCherry construct expressed detectable mCherry (data not shown). Immunoblotting analyses showed that cells transduced with hCSK-mCherry, denoted as CSK overexpressor (OE), exhibited, respectively, about two- to three-fold higher CSK levels than cells transduced with the empty pCDH vector (Figures S1E,F in Supplementary Material). To find out whether altering CSK expression by the transduction procedure and selection in puromycin has any effect on the appearance of FcɛRI and c-KIT receptors on the cell surface, we examined various cell types by flow cytometry. We found that cells transduced with individual CSK-specific shRNAs (data not shown), pooled CSK-specific shRNA (CSK-KD), or CSK-OE displayed an abundance of FcɛRI and c-KIT receptors on the cell surface comparable to corresponding control cells transduced with empty vectors pLKO.1 or pCDH (Figures S1G,H in Supplementary Material).

### Enhanced Degranulation, Calcium Response, and Migration, but Reduced Adhesion to Fibronectin and FcεRI–IgE Complex Internalization in BMMCs With CSK-KD

**Figure 1:**
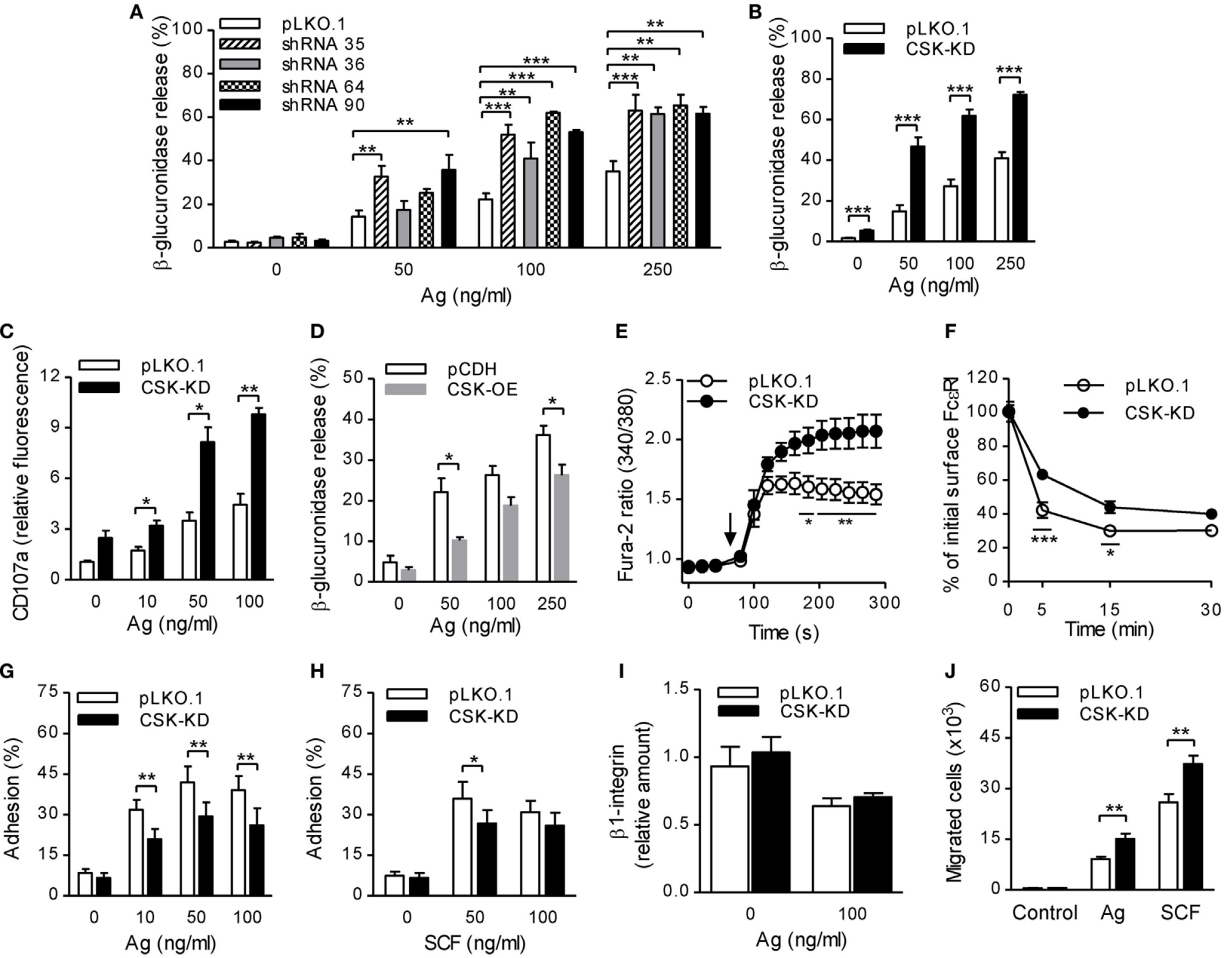
Enhanced degranulation, calcium response, and chemotaxis toward antigen and SCF, but reduced adhesion to fibronectin and FcεRI–IgE complexes internalization in bone marrow-derived mast cells (BMMCs) with C-terminal Src kinase (CSK)-KD. **(A)** β-glucuronidase release was analyzed in BMMCs transduced with individual CSK-specific shRNAs (35, 36, 64, or 90) in pLKO.1 vector or empty pLKO.1 vector (pLKO.1). The cells were sensitized with IgE and activated with various concentrations of antigen (Ag) for 30 min. The data represent means ± SEM from 4–8 independent experiments performed in duplicates or triplicates. **(B)** β-glucuronidase release from BMMCs transduced with a pool of shRNAs (CSK-KD) or empty pLKO.1 vector was examined as in **(A)**. The data represent means ± SEM from eight independent experiments performed in duplicates or triplicates. **(C)** Presence of CD107a on the cell surface in BMMCs with CSK-KD or empty pLKO.1 vector used as a control. The cells were sensitized with IgE, activated with various concentrations of antigen for 10 min and the presence of CD107a on the cell surface was analyzed by flow cytometry. The results shown are means ± SEM from three independent experiments performed in duplicates. **(D)** β-glucuronidase release from stable cell line derived from BMMCs transduced with empty vector (pCDH) or pCDH vector containing hCSK-mCherry construct (CSK-OE) was analyzed as in **(A)**. The data represent means ± SEM from three independent experiments performed in duplicates or triplicates. **(E)** Calcium response examined in BMMCs with CSK-KD and corresponding controls. The cells were sensitized with IgE, loaded with Fura-2, and activated with antigen (100 ng/ml) added as indicated by an arrow. Data represent means ± SEM calculated from six independent experiments each performed in duplicate. **(F)** IgE internalization in BMMCs with CSK-KD or control pLKO.1 cells. The IgE-sensitized cells were activated with antigen (500 ng/ml) for various time intervals and fixed with 4% paraformaldehyde. IgE was quantified using AF488-labeled anti-mouse IgG (IgE cross-reacting) antibody by flow cytometry. Means ± SEM calculated from three independent experiments are shown. **(G,H)** Cell adhesion to fibronectin-coated surfaces. BMMCs with CSK-KD or pLKO.1 control cells were sensitized with IgE, loaded with calcein, and activated with various concentrations of antigen **(G)** or SCF **(H)**. Fluorescence was determined before and after washing out non-adherent cells, and the percentages of adherent cells were calculated. The results indicate means ± SEM from five independent experiments. **(I)** The presence of β1-integrin on the cell surface in BMMCs with CSK-KD and corresponding control cells analyzed by flow cytometry. **(J)** BMMCs with CSK-KD or pLKO.1 control cells were sensitized with IgE and their migration toward antigen (250 ng/ml) or SCF (50 ng/ml) was determined. The data represent means ± SEM from five independent experiments each performed in duplicates. Statistical significance of intergroup differences was determined using one-way ANOVA with Tukey’s post-test **(A)** or unpaired two-tailed Student’s *t*-test **(B–D,G–J)** or two-way ANOVA with Bonferroni post-test **(E,F)**. *P < 0.05; **P < 0.01; and ***P < 0.001.

To find out whether reduced expression of CSK in BMMCs affects their FcεRI-mediated degranulation, the cells transduced with individual shRNAs (35, 36, 64, and 90) or pooled shRNAs were sensitized with IgE and activated with various concentrations of antigen. Degranulation was determined by quantification of β-glucuronidase released from the cells upon antigen exposure or spontaneously. In nonactivated cells, there were no significant changes in spontaneous β-glucuronidase released from the cells transduced with various individual shRNA vectors or control pLKO.1 (Figure 1A). After FcɛRI triggering with antigen at a concentration of 100 or 250 ng/ml, significantly increased degranulation was observed in all cell types transduced with various shRNAs, when compared to pLKO.1 control cells (Figure 1A). Significantly increased degranulation was also observed when cells were transduced with pooled shRNAs (Figure 1B). It should be noted that the total amount of β-glucuronidase present in all cell types was similar (data not shown), indicating a negative regulatory role of CSK on FcεRI-mediated degranulation, rather than on enhanced production of β-glucuronidase. Because the results obtained with cells transduced with individual shRNAs or pooled shRNAs were similar, we decided to combine the data and present them as data from cells with CSK-KD.

Previous studies showed that mast cell degranulation is accompanied by translocation of CD107a onto the cell surface (37). To determine whether CSK has any effect on this process, we examined the presence of CD107a on the cell surface by flow cytometry in BMMCs with CSK-KD and control cells before and after activation with antigen. The results show that BMMCs with CSK-KD activated for 10 min with various concentrations of antigen exhibited significantly higher presence of CD107a on the cell surface than cells transduced with pLKO.1 control vector (Figure 1C). To further confirm a negative regulatory role of CSK in mast cell activation, we next examined mast cell activation in cells overexpressing CSK (CSK-OE). The data presented in Figure 1D show that when activated with antigen (50 and 250 ng/ml) BMMCs with CSK-OE exhibited significantly decreased β-glucuronidase release when compared with control pCDH cells.

FcɛRI aggregation induces rapid Ca^2+^ mobilization, which is required for mast cell degranulation (38). To elucidate the role of CSK in Ca^2+^ signaling, IgE-sensitized cells with CSK-KD were loaded with intracellular Ca^2+^ indicator, Fura-2, and mobilization of Ca^2+^ was measured upon antigen stimulation. We found that Ca^2+^ levels were significantly increased in BMMCs with CSK-KD when compared to cells transduced with empty pLKO.1 vector (Figure 1E). To determine whether CSK interferes with FcεRI internalization, we used flow cytometry and measured the fraction of total IgE remaining on the cell surface at various time intervals after antigen-mediated aggregation of the FcεRI–IgE complexes. We found that upon antigen triggering, BMMCs with CSK-KD showed significantly reduced internalization of the antigen–IgE–FcεRI complexes when compared to pLKO.1 control cells (Figure 1F). The combined data indicate that CSK in BMMCs is a negative regulator of FcεRI-mediated degranulation and calcium response, while FcεRI internalization is positively regulated by CSK.

### BMMCs With CSK-KD Exhibit Reduced Adhesion to Fibronectin and Enhanced Chemotactic Response

Next, we examined the role of CSK in adhesion of antigen- or SCF-activated BMMCs to fibronectin-coated wells. Upon activation with various concentrations of antigen, BMMCs with CSK-KD exhibited significantly lower adhesion to fibronectin than control cells at all antigen concentrations tested (Figure 1G). When the CSK-KD cells were activated by SCF, less dramatic reduction of adhesion to fibronectin was observed; the difference was significant when SCF at a concentration 50 ng/ml was used (Figure 1H). It should be noted that the reduced adhesion of cells with CSK-KD was not caused by changes in β1-integrin abundance on the cell surface. We found that upon FcɛRI-mediated activation, amount of the cell surface exposed β1-integrin decreased in both BMMCs with CSK-KD or pLKO.1 controls and no differences between these two cell types were noticed (Figure 1I). An important feature of mast cells is their ability to migrate toward chemoattractants, which can be simulated by an *in vitro* transwell-migration assay (39). When compared to control cells, BMMCs with CSK-KD exhibited enhanced migration toward antigen as well as toward SCF, which is a more potent chemoattractant than antigen (Figure 1J). Altogether, these data indicate that CSK in BMMCs is a positive regulator of adhesion to fibronectin and negative regulator of migration toward antigen and SCF.

### Negative and Positive Regulatory Roles of CSK on Tyrosine Phosphorylation of Early Signal-Transduction Molecules

**Figure 2:**
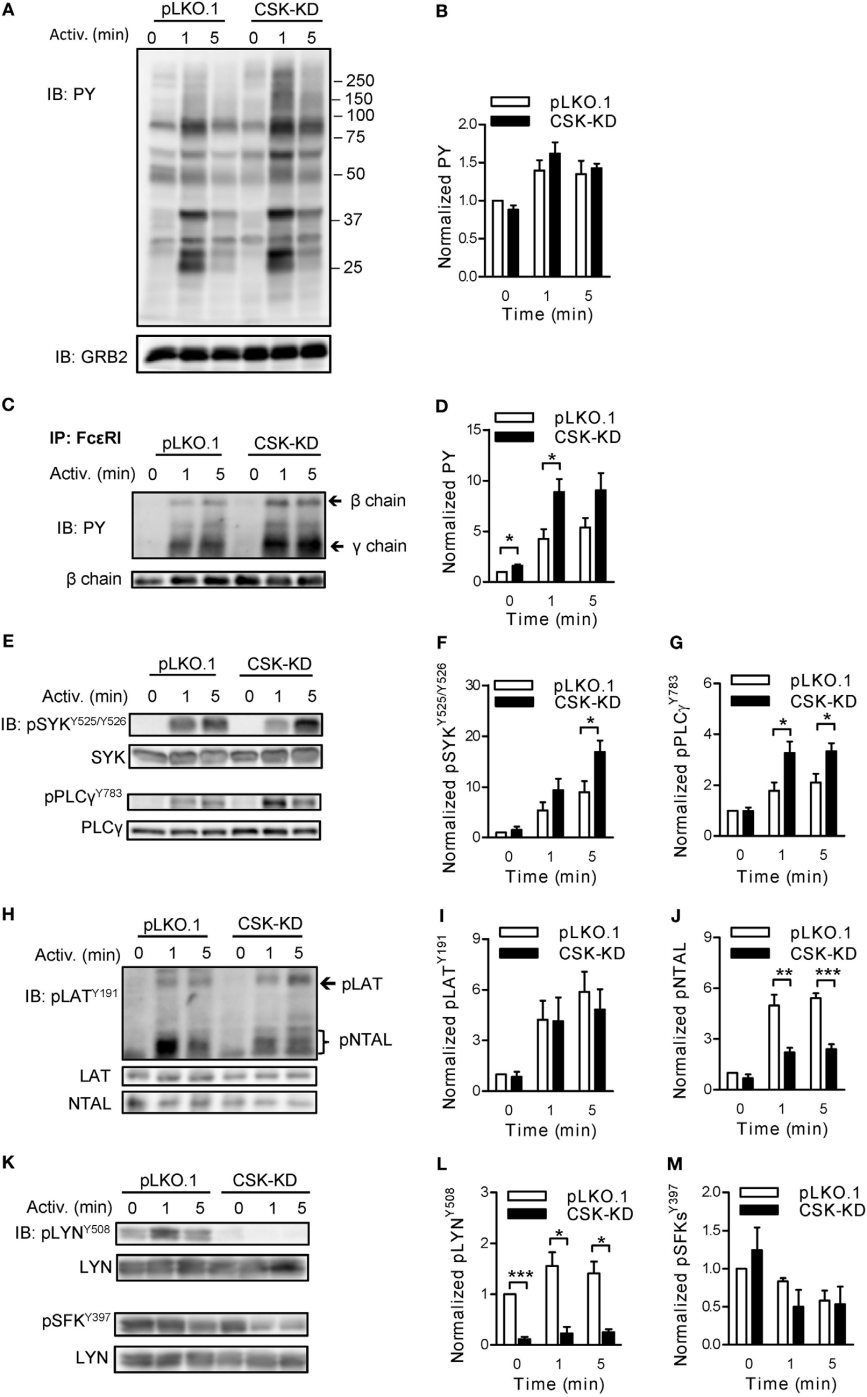
Negative and positive regulatory roles of C-terminal Src kinase (CSK) in tyrosine phosphorylation of early signal-transduction molecules. **(A)** Total tyrosine phosphorylation of IgE-sensitized bone marrow-derived mast cells (BMMCs) with CSK-KD or control pLKO.1 cells activated or not with antigen (250 ng/ml) for various time intervals. The cells were lysed and analyzed by immunoblotting (IB) with tyrosine-specific monoclonal antibodies PY20-HRP conjugate (PY). GRB2 was used as a loading control. Representative immunoblots from three experiments are shown. Numbers on the right indicate positions of molecular weight standards. **(B)** Densitometry analysis of the immunoblots as from panel **(A)**. **(C)** FcɛRIs were immunoprecipitated (IP) from the lysates of nonactivated or antigen-activated BMMCs with CSK-KD or control cells (pLKO.1). The immunoprecipitates were examined by immunoblotting with PY20-HRP conjugate. For loading controls, FcɛRI β chain-specific antibody was used. Positions of FcɛRI β and γ chains are marked by arrows. A representative immunoblot from three performed is shown. **(D)** Densitometry analysis of the immunoblots as from panel **(C)**, in which pooled signals from tyrosine-phosphorylated FcεRI β and γ chains in activated cells were normalized to the signals from nonactivated cells and loading control protein (FcεRI β chain). Means ± SEM were calculated from three independent experiments. **(E–M)** IgE-sensitized BMMCs with CSK-KD or control pLKO.1 cells were activated with antigen for various time intervals and whole-cell lysates were analyzed by immunoblotting for tyrosine phosphorylated SYK [pSYK^Y525/Y526^; **(E,F)**], and PLCγ [pPLCγ^783^; **(E,G)**]. Tyrosine phosphorylated LAT [pLAT^Y191^; **(H,I)**] was analyzed with anti-LAT^Y191^ antibody, which recognizes also phosphorylated NTAL [pNTAL; **(H,J)**]. Tyrosine phosphorylated LYN [pLYN^Y508^; **(K,L)**] and pSFKs^Y397^ **(K,M)** were analyzed with the corresponding antibodies. Representative immunoblots for each phosphorylated protein with corresponding loading controls are shown **(E,H,K)**. The results in **(F,G,I,J,L,M)** show densitometry analysis of the corresponding immunoblots in which signals from tyrosine-phosphorylated proteins in activated cells were normalized to the signals in nonactivated cells and loading control proteins. Means ± SEM were calculated from 4–7 independent experiments. Statistical significance of differences between CSK-KD and pLKO.1 cells was determined using unpaired two-tailed Student’s *t*-test. *P < 0.05; **P < 0.01; and ***P < 0.001.

To contribute to understanding the molecular mechanism by which CSK regulates degranulation and Ca^2+^ response after FcɛRI triggering, we first examined the tyrosine phosphorylation profiles of selected signal-transduction proteins. We found no dramatic differences in global tyrosine phosphorylation between nonactivated BMMCs with CSK-KD and control pLKO.1 cells (Figure 2A). One minute after antigen-mediated activation (250 ng/ml), control cells exhibited enhanced global tyrosine phosphorylation of several substrates, which was downregulated 5 min after triggering. A similar set of proteins showed enhanced phosphorylation in the cells with CSK-KD (Figures 2A,B).

Next we assessed the role of CSK in tyrosine phosphorylation of individual proteins involved in early signal-transduction events after FcɛRI triggering. BMMCs with CSK-KD and the corresponding control pLKO.1 cells were sensitized with IgE, activated or not with antigen (250 ng/ml) for 1 or 5 min, solubilized, and tyrosine phosphorylation of the immunoprecipitated FcɛRI chains was examined by immunoblotting. Data show that after antigen-mediated activation, FcεRI β and γ chains were rapidly tyrosine phosphorylated in both cell types (Figure 2C). However, cells with CSK-KD, when compared to control cells, exhibited significantly higher phosphorylation of the FcεRI β and γ chains in nonactivated state and 1 min after triggering (Figures 2C,D). Consistent with the increased degranulation and calcium response are findings of significantly increased tyrosine phosphorylation of SYK^Y525/Y526^ and PLCγ^Y783^ in antigen-activated cells with CSK-KD when compared to control cells (Figures 2E–G).

A previous study showed that inhibition of CSK enzymatic activity in T cells resulted in enhanced tyrosine phosphorylation of adaptor protein LAT (40). However, there are no data on the regulatory role of CSK toward another adaptor protein similar to LAT but absent in nonactivated T cells, NTAL, which is expressed together with LAT in mast cells (41). We, therefore, next examined tyrosine phosphorylation of both adaptor proteins in antigen-activated BMMCs with CSK-KD and controls using anti-phosphotyrosine LAT^Y191^ antibody that recognizes a common tyrosine-phosphorylated epitope in both LAT and NTAL (42). In nonactivated cells, tyrosine phosphorylation of LAT^Y191^ and NTAL was low and comparable in both cell types. When compared to corresponding controls, antigen-activated BMMCs with CSK-KD exhibited comparable tyrosine phosphorylation of the LAT^Y191^ adaptor protein but, surprisingly, significantly decreased tyrosine phosphorylation of the NTAL adaptor (Figures 2H–J).

Tyrosine phosphorylations of the FcεRI subunits and also LAT and NTAL are mediated, at least in part, by the LYN kinase activity (43). The enzymatic activity of LYN is positively regulated by auto-phosphorylation of its Y^397^ and negatively by CSK-mediated phosphorylation of its C-terminal Y^508^ (16). To determine the role of CSK in phosphorylation of these tyrosines, the cells were activated as above and analyzed by immunoblotting with the corresponding phosphotyrosine-specific antibodies. In control BMMCs, transduced with empty pLKO.1 vector, LYN^Y508^ was phosphorylated in nonactivated cells, and after activation its phosphorylation did not show significant changes. As expected, BMMCs with CSK-KD, when compared to controls, showed significantly reduced phosphorylation of LYN^Y508^ in both nonactivated and activated cells (Figures 2K,L). When auto-phosphorylation of SFKs, using pSFK^Y397^-specific antibody, was analyzed in nonactivated cells, no significant changes were observed between BMMCs with CSK-KD and control cells. After antigen-mediated activation, both cell types exhibited slightly decreased phosphorylation of SFKs^Y397^ and again, no significant difference was observed between them (Figures 2K,M). In accordance with this observation, tyrosine phosphorylation of immunoprecipitated LYN and FYN analyzed by immunoblotting with pSFK^Y397^-specific antibody revealed no significant difference in phosphorylation at Y397 of LYN (Figures S2A,B in Supplementary Material) as well as FYN (Figures S2C,D in Supplementary Material).

### Different Regulation of LYN, FYN, and FcεRI-Bound Kinase Activity by CSK

**Figure 3:**
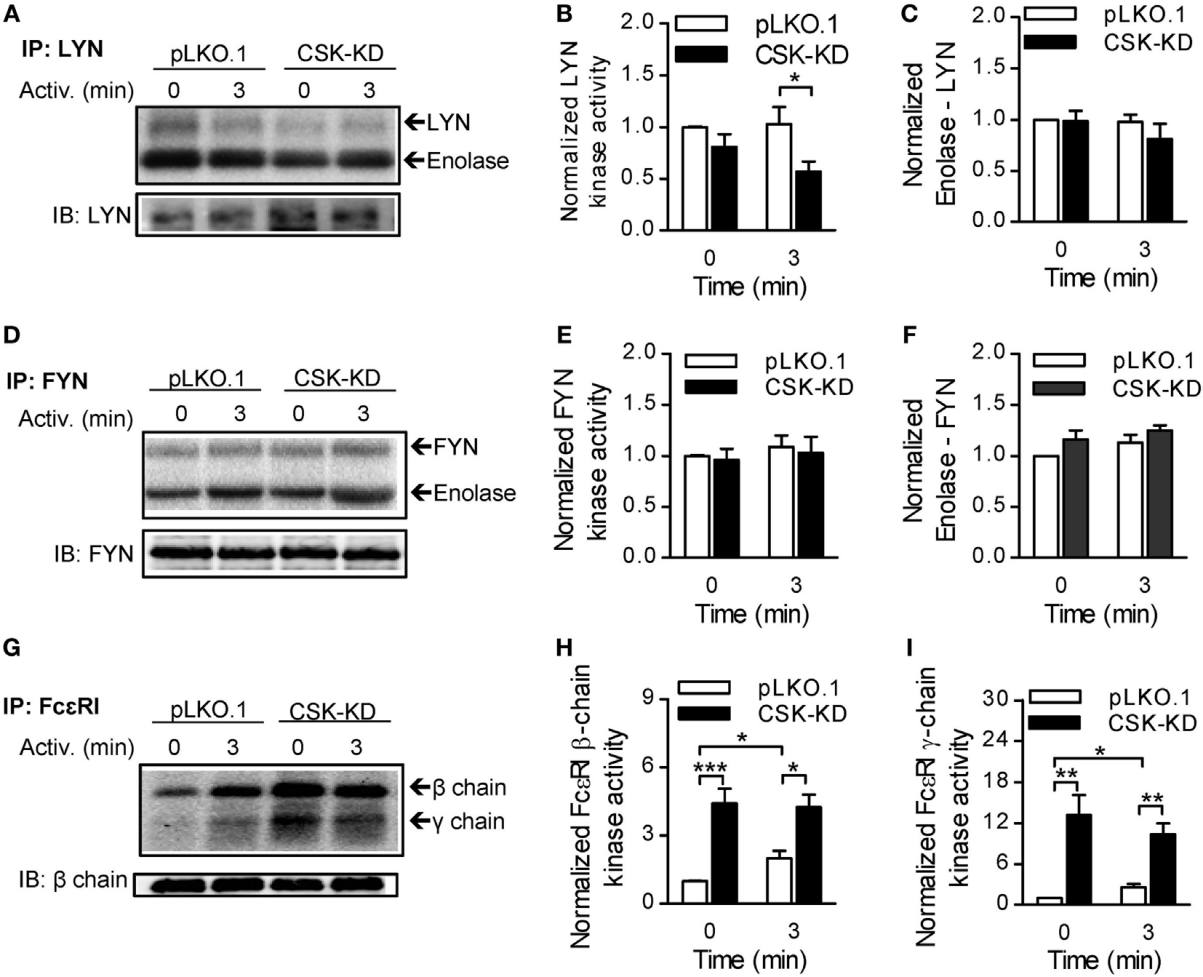
Different regulation of LYN, FYN, and FcεRI-bound kinase activity by C-terminal Src kinase (CSK). **(A)** LYN was immunoprecipitated from nonactivated or antigen-activated (250 ng/ml) BMMCs with CSK-KD or control cells (pLKO.1) and then incubated with kinase buffer containing [γ-^32^P]ATP and acid-denatured enolase used as an endogenous substrate. ^32^P-labeled proteins were size-fractionated, transferred to nitrocellulose membranes, and examined by autoradiography. **(B,C)** Signals from autoradiograms were quantified, and those corresponding to LYN **(B)** and enolase **(C)** were normalized to the amount of precipitated LYN and to the signals in nonactivated pLKO.1 BMMCs. **(D)** The kinase activity of immunoprecipitated FYN was determined as above, except that anti-FYN antibody was used for immunoprecipitation. **(E,F)** Signals from autoradiograms of FYN **(E)** and enolase **(F)** were quantified and normalized to the amount of precipitated FYN and to the signals in nonactivated control cells. **(G)** The kinase activity bound to immunoprecipitated FcεRI was determined using a kinase assay in which anti-IgE was used for IgE–FcεRI complex precipitation, and phosphorylation of FcεRI β and γ chains was examined. **(H,I)** Signals from autoradiograms of FcεRI β chain **(H)** and FcεRI γ chain **(I)** were quantified and normalized to the amount of precipitated FcεRI β chain and to the signals in nonactivated control cells. The means ± SEM were calculated from five independent experiments in each panel. Statistical significance of intergroup differences was determined using unpaired two-tailed Student’s *t*-test. *P < 0.05; **P < 0.01; and ***P < 0.001.

The observed changes in phosphorylation of signal-transduction molecules between BMMCs with CSK-KD and the corresponding controls led us to compare the regulatory effects of CSK on LYN kinase activity in nonactivated and antigen-activated cells. For these experiments, LYN was immunoprecipitated, and its auto-phosphorylation and phosphorylation of the exogenous substrate, an acid-denaturated enolase, was examined by an *in vitro* kinase assay. LYN kinase from nonactivated BMMCs with CSK-KD and control pLKO.1 cells exhibited comparable enzymatic activities as reflected by similar auto-phosphorylation and enolase phosphorylation. However, upon antigen stimulation, immunoprecipitated LYN from cells with CSK-KD showed lower auto-phosphorylation but no significant difference in phosphorylation of enolase when compared to LYN from the control cells (Figures 3A–C).

In a kinase assay, we also assessed the enzymatic activity of FYN kinase, which is an important regulator of FcεRI-induced degranulation (44), and found no difference in auto-phosphorylation and enolase phosphorylation between FYN immunoprecipitated from nonactivated or antigen-activated BMMCs with CSK-KD or control cells (Figures 3D–F).

Finally, we examined phosphorylation of FcɛRI subunits by the FcεRI-associated kinases. The FcεRI–IgE complexes were isolated by immunoprecipitation from nonactivated or antigen-activated BMMCs with CSK-KD or control cells and examined by *in vitro* kinase assays. In nonactivated cells, FcεRI β and γ subunits showed significantly higher phosphorylation when derived from BMMCs with CSK-KD than from control cells. After activation, there was a significant increase in phosphorylation of the receptor subunits from control cells, but no significant change when the receptor was obtained from cells with CSK-KD (Figures 3G–I). These data are in accord with tyrosine phosphorylation of the FcɛRI subunits as detected by immunoblotting (Figures 2C,D).

### BMMCs With CSK-KD Exhibit Reduced Cytokine and Chemokine Production, Reduced STAT5 Tyrosine Phosphorylation, and Enhanced Tyrosine Phosphorylation of SHP-1

**Figure 4:**
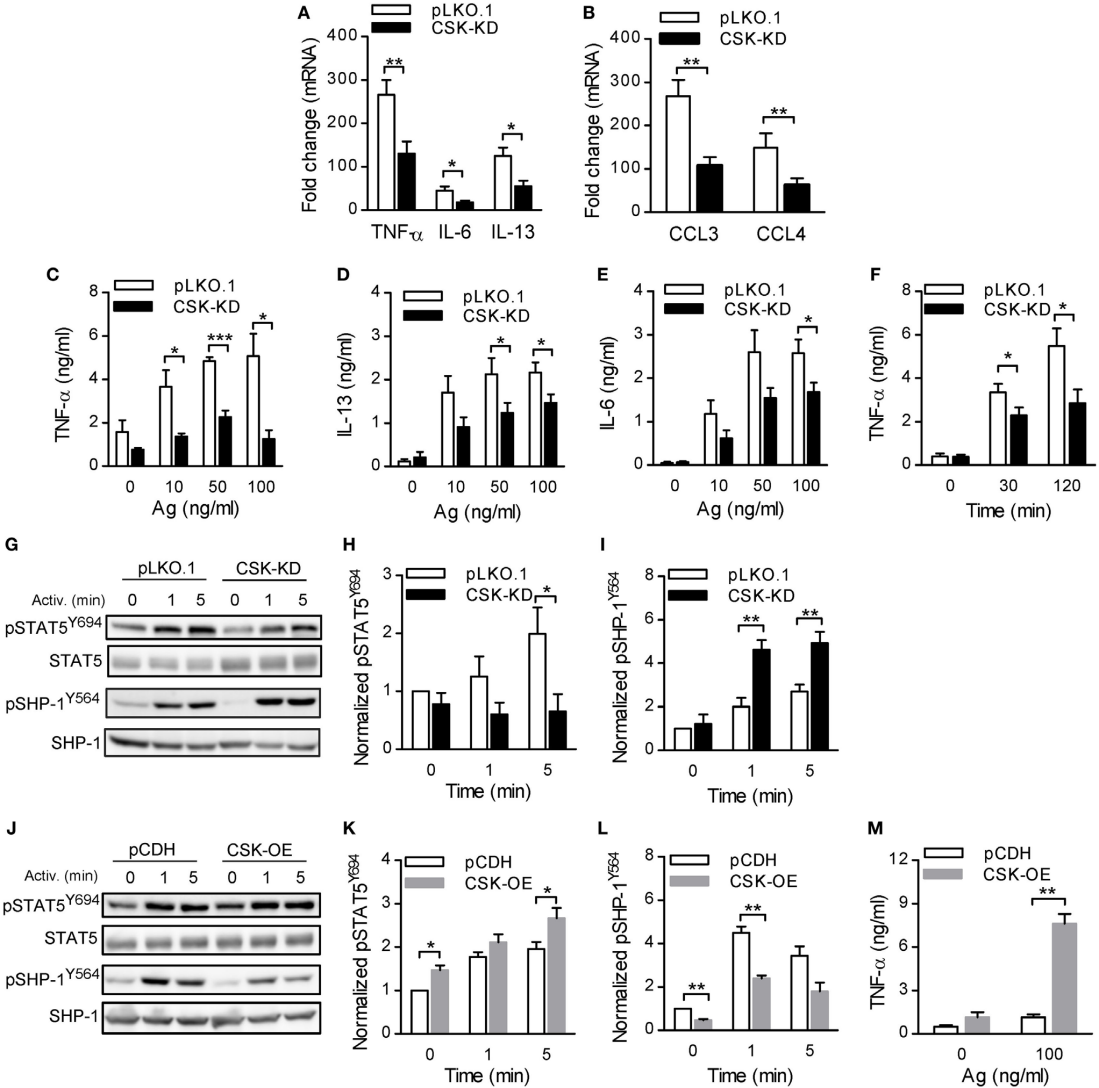
Activated bone marrow-derived mast cells (BMMCs) with C-terminal Src kinase (CSK)-KD exhibit reduced cytokine and chemokine production, reduced STAT5 tyrosine phosphorylation, and enhanced SHP-1 tyrosine phosphorylation. **(A,B)** IgE-sensitized BMMCs with CSK-KD or pLKO.1 control cells were activated for 1 h with antigen (100 ng/ml), and mRNAs encoding cytokines (TNF-α, IL-6, and IL-13); **(A)** or chemokines (CCL3 and CCL4); **(B)** were quantified by RT-qPCR. Means ± SEM were calculated from four independent experiments. **(C–E)** IgE-sensitized BMMCs with CSK-KD or pLKO.1 control cells were activated or not for 6 h with various concentrations of antigen and the concentrations of cytokines TNF-α **(C)**, IL-13 **(D)**, and IL-6 **(E)** secreted into the supernatants were determined. Means ± SEM were calculated from 4–8 independent experiments performed in duplicates or triplicates. **(F)** The cells were analyzed as in **(C)** except that they were activated with only one concentration of antigen (50 ng/ml) for 30 or 120 min. Means ± SEM were calculated from four independent experiments performed in duplicates. **(G)** Representative immunoblots of whole-cell lysates from BMMCs with CSK-KD or pLKO.1 control cells activated for 1 or 5 min or not activated with antigen and analyzed by immunoblotting with antibodies to tyrosine-phosphorylated STAT5 (pSTAT5^Y694^) and SHP-1 (pSHP-1^Y564^). For loading controls, the membranes were analyzed by immunoblotting for STAT5 and SHP-1. **(H,I)** Densitometry analysis of the immunoblots in which signals from pSTAT5^Y694^ **(H)** and pSHP-1^Y564^ **(I)** in activated cells were normalized to the signals from nonactivated control cells and loading control proteins. Means ± SEM were calculated from 4–6 experiments. **(J)** Representative immunoblots of whole-cell lysates from BMMCs with CSK-OE or pCDH control cells, activated and analyzed as described in Figure 3G, are shown. **(K,L)** Densitometry analysis of the immunoblots was performed as in Figures 3H,I. Means ± SEM were calculated from three independent experiments. **(M)** IgE-sensitized BMMCs with CSK-OE or pCDH control cells were activated or not with antigen and concentrations of TNF-α secreted into the supernatants were determined. Means ± SEM were calculated from two independent experiments performed in duplicates or triplicates. Statistical significance of intergroup differences was determined using unpaired two-tailed Student’s *t*-test. *P < 0.05; **P < 0.01; and ***P < 0.001.

Production and release of cytokines and chemokines is an important feature of the FcεRI-induced mast cell activation (1). In further experiments we, therefore, compared these responses between BMMCs with CSK-KD and pLKO.1 control cells. IgE-sensitized cells were activated with antigen, and mRNA levels of various cytokines (TNF-α, IL-6, and IL-13) and chemokines (CCL3 and CCL4) were analyzed by RT-qPCR. Interestingly, the mRNA levels of all studied cytokines (Figure 4A) and chemokines (Figure 4B) were significantly reduced in BMMCs with CSK-KD when compared to control cells. Next, we measured secretion of the cytokines into the media. In nonactivated cells, there were no significant differences in basal levels of secreted cytokines between BMMCs with CSK-KD and control cells. Upon 6 h stimulation with various concentrations of antigen, the concentrations of secreted TNF-α (Figure 4C), IL-13 (Figure 4D), and IL-6 (Figure 4E) were significantly lower in BMMCs with CSK-KD than in control cells. It is known that TNF-α is an early secreted mediator in antigen-activated cells (45, 46). Therefore, we also examined secretion of TNF-α at earlier time points and found significantly reduced TNF-α secretion into the media 30 and 120 min after antigen (50 ng/ml) triggering in BMMCs with CSK-KD when compared to control cells (Figure 4F).

To understand the mechanism how CSK regulates FcεRI-induced cytokine and chemokine production, we examined tyrosine phosphorylation of transcription factor STAT5, which is one of the key regulators of cytokine production in mast cells (47). Indeed, STAT5^Y694^ tyrosine phosphorylation in antigen-activated CSK-KD cells was significantly decreased when compared to control cells (Figures 4G,H). Previous studies have shown that the activity of STAT5 is negatively regulated by SHP-1 (48) and that phosphorylation of SHP-1 at tyrosine 564 is indispensable for its maximal phosphatase activity (49). To investigate the molecular mechanism responsible for the decreased STAT5 tyrosine phosphorylation in CSK-KD cells we analyzed phosphorylation of SHP-1 at tyrosine 564 (SHP-1^Y564^) in the course of FcεRI-mediated activation by immunoblotting. In nonactivated cells, there was only weak tyrosine phosphorylation of this phosphatase, and CSK had no significant effect on it. Interestingly, phosphorylation of SHP-1^Y564^ was significantly enhanced in antigen-stimulated CSK-KD cells when compared to control pLKO.1 cells (Figures 4G,I). To determine whether CSK is involved in STAT5 and SHP-1 regulation, we also analyzed mast cells with enhanced expression of CSK. We found that cells with CSK-OE exhibited increased tyrosine phosphorylation of STAT5^Y694^ not only upon FcεRI triggering but also in nonactivated cells (Figures 4J,K). Consistent with the increased phosphorylation of STAT5^Y694^, we observed significantly reduced phosphorylation of SHP-1^Y564^ both in nonactivated and antigen-activated mast cells with CSK-OE when compared to cells transduced with pCDH control vector (Figures 4J,L). Finally, we examined secretion of TNF-α. IgE-sensitized mast cells with CSK-OE and cells transduced with pCDH control vector were stimulated with antigen or not, and TNF-α secreted into the media was quantified. In nonactivated cells, there were no significant differences in the basal levels of secreted TNF-α between mast cells with CSK-OE and controls. Upon stimulation with antigen, the concentrations of secreted TNF-α were significantly increased in mast cells with CSK-OE when compared to controls (Figure 4M). Altogether, these data indicate that CSK in BMMCs is a positive regulator of proinflammatory cytokines and chemokines production.

### LYN Is Indispensable for Tyrosine Phosphorylation of SHP-1^Y564^ in Mast Cells

**Figure 5:**
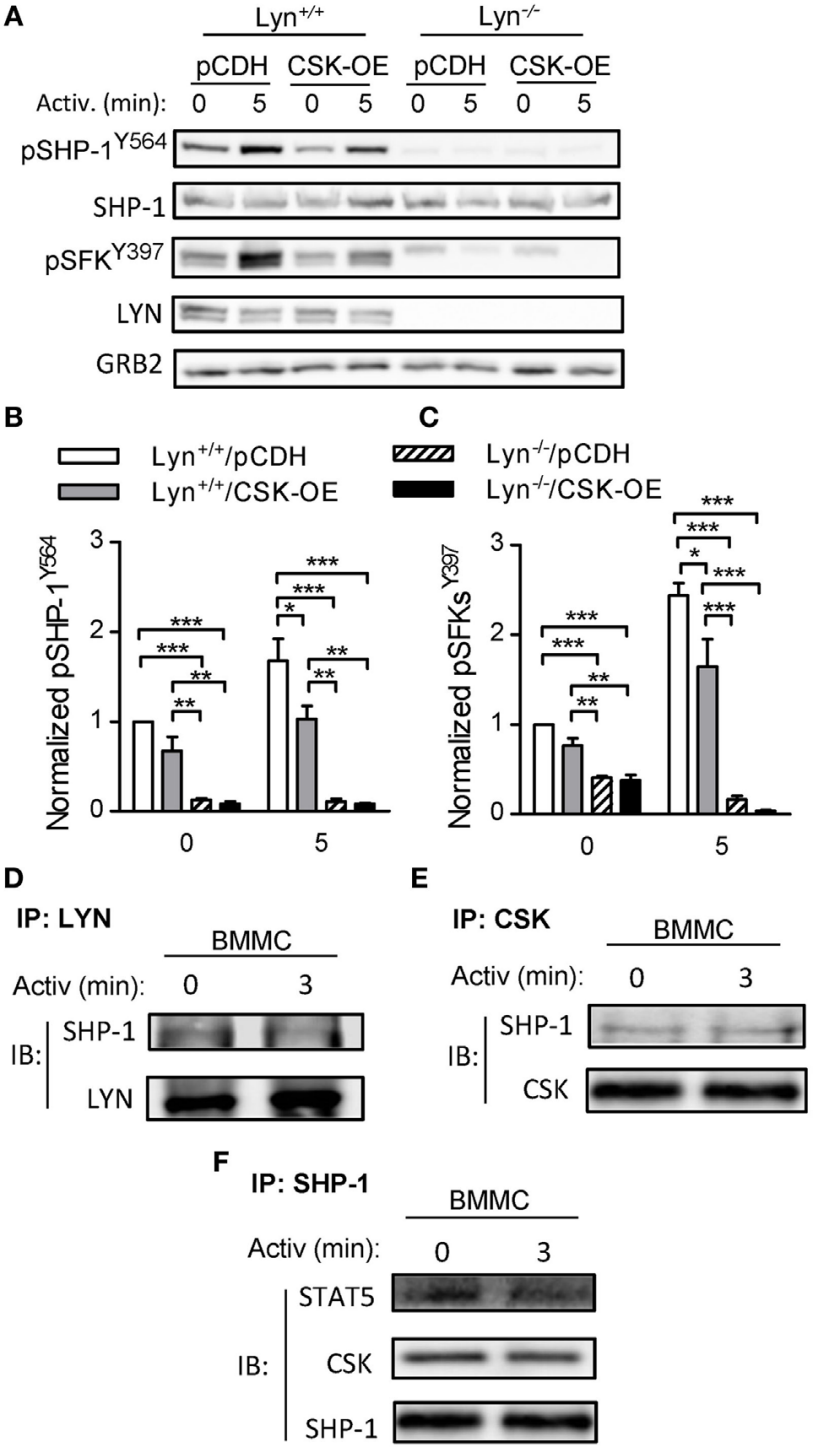
Tyrosine phosphorylation of SHP-1^Y564^ and pSFK^Y397^ in Lyn^+/+^ and Lyn^−/−^ bone marrow-derived mast cells (BMMCs) lines with C-terminal Src kinase (CSK)-OE and controls and physical interactions among CSK, LYN, SHP-1, and STAT5 in WT BMMCs. **(A)** IgE-sensitized Lyn^+/+^ or Lyn^−/−^ stable BMMC lines with CSK-OE or control pCDH were activated or not with antigen (250 ng/ml) and whole-cell lysates were analyzed by immunoblotting for tyrosine phosphorylated SHP-1^Y564^ (pSHP-1^Y564^) and Src family tyrosine kinases (SFKs) (pSFKs^Y397^). For loading controls, SHP-1-specific, LYN-specific, and GRB-specific antibodies were used. **(B)** Densitometry analysis of the immunoblots as in panel **(A)**, in which signals from tyrosine-phosphorylated SHP-1^Y564^ were normalized to the signals from nonactivated Lyn^+/+^/pCDH control cells and loading control protein (SHP-1). **(C)** Densitometry analysis of tyrosine-phosphorylated SFKs (pSFKs^Y397^) performed as in **(B)** with GRB2 as a loading control protein. **(D–E)** Coimmunoprecipitation experiments. **(D)** Nonactivated or antigen-activated BMMCs were lysed and immunoprecipitated with an antibody specific for LYN kinase. The immunocomplexes were examined by immunoblotting with antibody specific for SHP-1. LYN-specific antibody was used as a loading control. **(E)** Cell lysates were obtained as in **(D)** and immunoprecipitated with antibody specific for CSK. The immunocomplexes were examined by immunoblotting with protein-specific antibodies to determine presence of SHP-1 and CSK, used as a loading control. **(F)** Cell lysates were obtained as in **(D)** and immunoprecipitated with an antibody specific for SHP-1. The immunocomplexes were examined by immunoblotting with protein-specific antibodies to determine presence of CSK and STAT5; SHP-1 was used as a loading control. Representative data from three independent experiments are shown in **(A,D–F)**. Means ± SEM in **(B,C)** were calculated from three independent experiments. Statistical significance of intergroup differences in **(B,C)** was determined using one-way ANOVA with Tukey’s post-test. *P < 0.05; **P < 0.01; and ***P < 0.001.

C-terminal Src kinase is considered to be a major negative regulator of SFKs. To determine whether CSK and LYN are involved in regulation of tyrosine phosphorylation of SHP-1 in mast cells we examined properties of stable BMMC line deficient in LYN (Lyn^−/−^) with CSK-OE (Lyn^−/−^/CSK-OE) and compared it with WT BMMC line with CSK-OE (Lyn^+/+^/CSK-OE) and the corresponding control cells (Lyn^+/+^/pCDH; Lyn^−/−^/pCDH). As shown in Figures 5A,B, tyrosine phosphorylation of SHP-1^Y564^ was observed in non-activated control Lyn^+/+^ cells and after antigen triggering the phosphorylation was increased. In Lyn^+/+^/CSK-OE cells phosphorylation of pSHP-1^Y564^ was significantly reduced in antigen-activated cells. In Lyn^−/−^ cells reduced phosphorylation of SHP-1^Y564^ was observed in both cell types, with CSK-OE or control pCDH vector. This result is consistent with enhanced tyrosine phosphorylation of SHP-1^Y564^ observed in BMMCs with CSK-KD (Figures 4G,I). Further experiments showed that decreased tyrosine phosphorylation of SHP-1^Y564^ correlated with reduced tyrosine phosphorylation of SFK^Y397^ in Lyn^+/+^ cells with CSK-OE (Figures 5A,C). Collectively, these data suggest that CSK regulates SHP-1^Y564^ phosphorylation through LYN activity.

### Physical Interaction Among CSK, LYN, SHP-1, and STAT5 in BMMCs

To support our hypothesis that there is a physical and functional interaction among CSK, LYN, SHP-1, and STAT5 we next examined immunocomplexes obtained from nonactivated or antigen-activated BMMCs by immunoprecipitation with antibodies specific for LYN, CSK, and SHP-1. We found that LYN immunocomplexes as well as CSK immunocomplexes from nonactivated as well as antigen-activated cells possessed SHP-1 (Figures 5D,E). Previous studies showed that SHP-1 physically interacts with STAT5 and that STAT5 activity is negatively regulated by SHP-1-mediated dephosphorylation (48, 49). To determine whether SHP-1 forms complexes with STAT5 we also examined SHP-1 immunoprecipitates. We found that SHP-1 immunocomplexes from nonactivated as well as antigen-activated BMMCs contain STAT-5 (Figure 5F). Further analysis indicated that the SHP-1 immunocomplexes also possess CSK. These data support our hypothesis that CSK could modulate activity of STAT5 through the LYN–SHP-1–STAT5 signaling axis.

### Production of PAG-KO BMMCs With Reduced or Enhanced CSK Expression

Previous studies have shown that CSK is anchored to the plasma membrane by TRAP PAG (4, 5). Interestingly, cells with PAG-KO or PAG-KD exhibited damping of FcɛRI-mediated mast cell activation both *in vitro* and *in vivo* (24). Thus, the properties of BMMC with PAG-KO or PAG-KD differed from those with CSK-KD described in this study. However, because PAG interacts with many other signal-transduction molecules (22), we decided to examine the properties of PAG-deficient mast cells with reduced expression of CSK. To this end, we used BMMCs from PAG-KO mice and produced CSK-KD in them by lentiviral-mediated delivery of CSK-specific shRNA (pool of four shRNAs) or empty pLKO.1 vector, followed by selection of puromycin-resistant clones, as described in Section “Materials and Methods.” Immunoblotting and densitometry analyses of such cells showed decreased expression of CSK by approximately 80% when compared to control cells transduced with empty pLKO.1 vector (Figures S3A,B in Supplementary Material). To find out whether altering CSK expression in BMMCs with PAG-KO has any effect on the presence of FcεRI and c-KIT receptors on the cell surface, we examined all transduced cell types and found no significant changes in the surface presence of FcɛRI and c-KIT (Figures S3C,D in Supplementary Material).

### BMMCs With CSK-KD and PAG-KO Exhibit Differences in FcεRI-Mediated Signaling Events When Compared to WT Cells or Cells With PAG-KO Alone

Our previous study showed that BMMCs isolated from mice with PAG-KO exhibited significantly reduced tyrosine phosphorylation of several molecules involved in early FcεRI-mediated signaling, including FcɛRI β and γ chains, SYK, LAT, and pPLCγ. This caused reduced degranulation and Ca^2+^ mobilization after antigen-mediated activation of PAG-KO BMMCs (24). On the other hand, this study shows that degranulation and Ca^2+^ responses were enhanced in cells with CSK-KD (Figures 1B,E). To better understand the regulatory roles and cross-talks between CSK and PAG during FcεRI-mediated mast cell signaling, we first compared degranulation and Ca^2+^ response in BMMCs with PAG-KO together with CSK-KD (denoted as CSK-KD/PAG-KO), control PAG-KO cells transduced with empty pLKO.1 vector (pLKO.1/PAG-KO), and control WT cells transduced with empty pLKO.1 vector (pLKO.1/WT).

**Figure 6:**
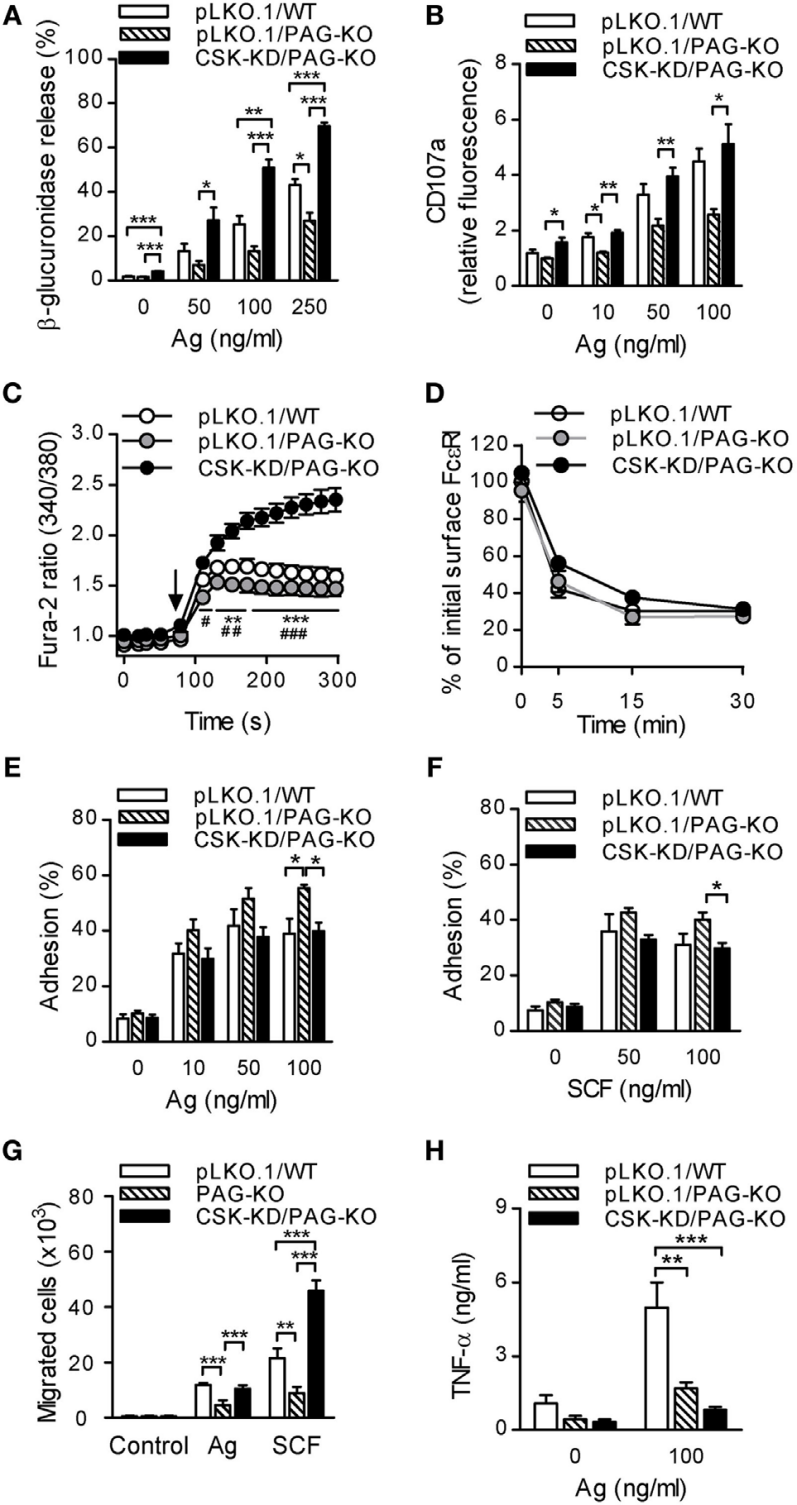
PAG-KO bone marrow-derived mast cells (BMMCs) with CSK-KD exhibit enhanced degranulation, calcium response, and migration toward chemoattractants, but reduced adhesion to fibronectin and production of TNF-α. **(A)** β-glucuronidase release was determined in IgE-sensitized PAG-KO BMMCs with CSK-KD (CSK-KD/PAG-KO) or pLKO.1/PAG-KO or pLKO.1/WT control cells activated (30 min) or not with various concentrations of antigen. **(B)** The presence of CD107a on the cell surface was analyzed by flow cytometry in IgE-sensitized and antigen-activated (10 min) CSK-KD/PAG-KO BMMCs and appropriate controls. **(C)** Calcium response examined in CSK-KD/PAG-KO BMMCs or pLKO.1/PAG-KO or pLKO.1/WT control cells. IgE-sensitized cells were loaded with Fura-2, exposed to antigen (100 ng/ml; arrow), and Fura-2 fluorescence was analyzed for 300 s. Statistical significance of differences between CSK-KD/PAG-KO versus pLKO.1/PAG-KO is denoted by asterisks and between CSK-KD/PAG-KO versus pLKO.1/WT are denoted by hashtags. The data in **(A–C)** represent means ± SEM calculated from 4–7 independent experiments performed in duplicates or triplicates. **(D)** IgE internalization in PAG-KO BMMCs with CSK-KD or control pLKO.1 cells. The IgE-sensitized cells were activated with antigen (500 ng/ml) for various time intervals and fixed with 4% paraformaldehyde. IgE was quantified as described in Figure 1F. Means ± SEM calculated from three independent experiments are shown. **(E,F)** Adhesion of CSK-KD/PAG-KO BMMCs or control cells to fibronectin-coated surfaces. The cells were sensitized with IgE, loaded with calcein and activated with various concentrations of antigen **(E)** or SCF **(F)**. Fluorescence was determined before and after washing out non-adherent cells, and the percentages of adherent cells were calculated. **(G)** Chemotaxis of BMMCs with PAG-KO, CSK-KD/PAG-KO, or wild-type (WT) cells was determined in Boyden chambers. The cells were sensitized with IgE and their migration toward antigen (250 ng/ml) and SCF (50 ng/ml) was determined. The results in E, F, and G represent means ± SEM from five independent experiments. **(H)** IgE-sensitized BMMCs with CSK-KD/PAG-KO or pLKO.1/PAG-KO or pLKO.1/WT control cells were activated or not for 6 h with antigen (100 ng/ml) and concentrations of TNF-α secreted into the supernatants were determined. Means ± SEM were calculated from four independent experiments. Statistical significance of intergroup differences was determined using one-way ANOVA with Tukey’s post-test **(A,B,E–H)** or two-way ANOVA with Bonferroni post-test **(C,D)**. * and ^#^*P* < 0.05; ** and ^##^*P* < 0.01; and *** and ^###^*P* < 0.001.

In the first series of experiments all cell types were sensitized with IgE and then activated with various concentrations of antigen. The results showed that nonactivated cells with CSK-KD/PAG-KO exhibited enhanced basal level of β-glucuronidase release when compared to pLKO.1/PAG-KO or pLKO.1/WT control cells (Figure 6A). Upon stimulation with antigen, CSK-KD/PAG-KO cells exhibited significantly elevated levels of β-glucuronidase at all antigen concentrations tested when compared to the control cells pLKO.1/PAG-KO and/or pLKO.1/WT (Figure 6A). Next, the surface localization of CD107a in antigen-activated or nonactivated cells was examined. Consistent with the β-glucuronidase release, significantly increased surface localization of CD107a after antigen activation was observed in CSK-KD/PAG-KO cells when compared to pLKO.1/PAG-KO controls (Figure 6B). In accord with our previous study (24) we found that antigen-activated control pLKO.1/PAG-KO cells exhibited decreased β-glucuronidase response and surface localization of CD107a when compared to pLKO.1/WT cells. Further, we analyzed the Ca^2+^ response. The cells were sensitized with IgE, loaded with Fura-2, and activated with antigen. We found that in the BMMCs with CSK-KD/PAG-KO, the Ca^2+^ response was significantly higher when compared to the Ca^2+^ response in control pLKO.1/PAG-KO and pLKO.1/WT cells (Figure 6C). On the other hand, there were no significant differences in internalization of the antigen–IgE–FcεRI complexes when compared CSK-KD/PAG-KO to pLKO.1 WT or PAG-KO control cells (Figure 6D).

Next, we examined adhesion of antigen-activated cells to fibronectin-coated wells. We found that BMMCs with CSK-KD/PAG-KO exhibited significantly reduced adhesion to fibronectin than pLKO.1/PAG-KO cells when antigen (Figure 6E) or SCF (Figure 6F) were used at a concentration 100 ng/ml. The extent of adhesion of CSK-KD/PAG-KO was comparable with adhesion of pLKO.1/WT cells. To determine the cross-talk between PAG and CSK in chemotactic response, in transwell-migration assay we examined migration of BMMCs with normal levels of PAG and CSK (WT), PAG-KO, and CSK-KD/PAG-KO toward antigen or SCF (Figure 6G). There were no differences among the cell types in the absence of chemoattractant. In cells exposed to antigen, PAG-KO cells exhibited reduced chemotaxis, as expected (24), while CSK-KD/PAG-KO cells exhibited enhanced migration toward antigen in comparison with the cells possessing PAG-KO alone. When chemotaxis toward SCF was examined, the migration of cells with CSK-KD/PAG-KO was significantly higher than that of WT and PAG-KO cells (Figure 6G). It should be noted that WT cells showed similar migration as cells transduced with the empty pLKO.1 vector [(24) and unpublished]. Finally, we examined production of TNF-α in nonactivated and antigen-activated (100 ng/ml) BMMCs. Our previous results (24) and data presented in Figure 6H in this study showed significantly decreased antigen-induced secretion of TNF-α in pLKO.1/PAG-KO cells when compared to pLKO.1/WT cells. Furthermore, we found that antigen-stimulated CSK-KD/PAG-KO cells exhibited even more pronounced inhibition of TNF-α secretion when compared to pLKO.1/WT cells, indicating positive regulatory role of CSK in BMMCs cytokine production (Figure 6H). The data also suggest that both PAG and CSK are implicated in BMMC adhesion and chemotaxis, but in different pathways.

**Figure 7:**
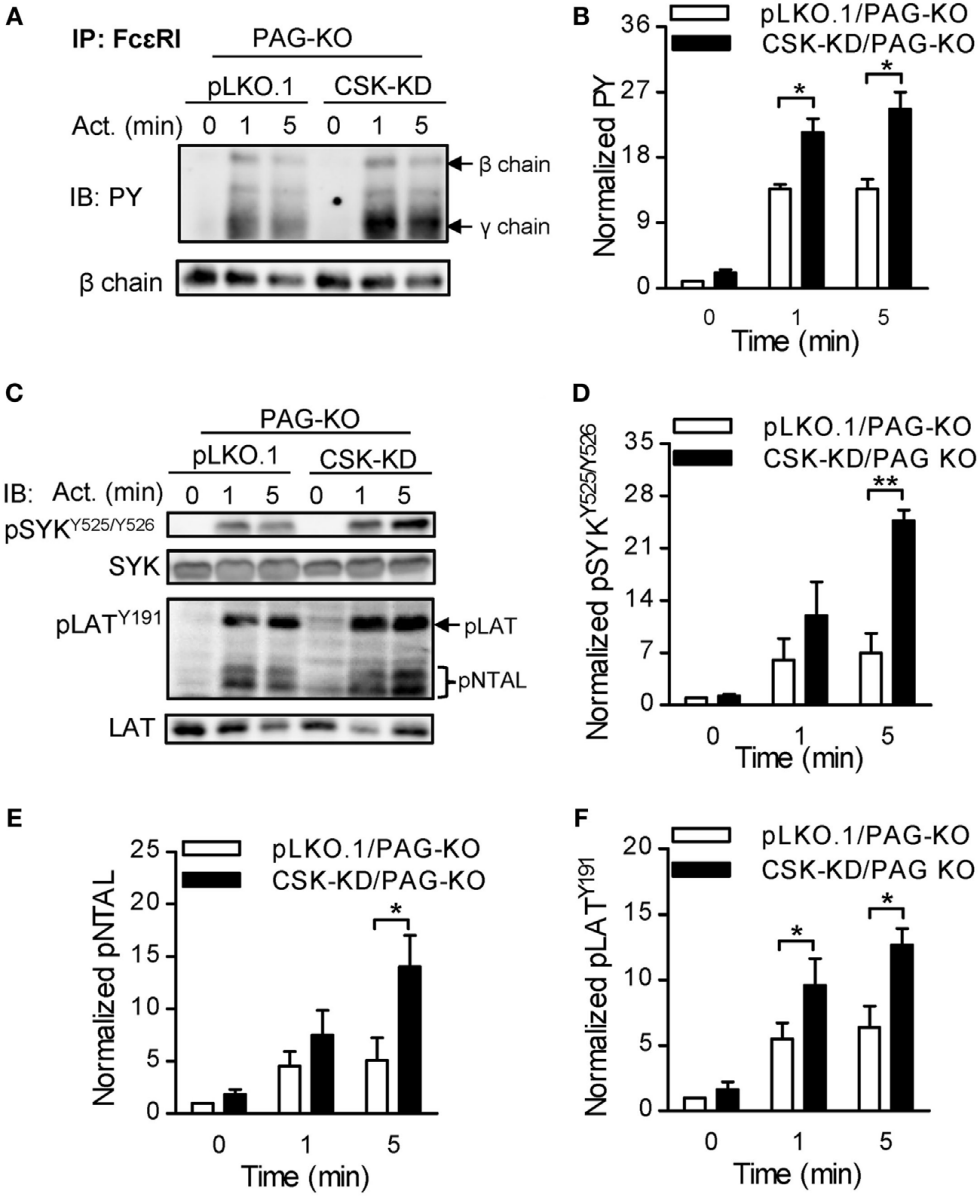
Enhanced tyrosine phosphorylation of early signal-transduction molecules in bone marrow-derived mast cells (BMMCs) with CSK-KD/PAG-KO. **(A)** FcɛRIs were immunoprecipitated from the lysates of CSK-KD/PAG-KO BMMCs or control cells activated with antigen (250 ng/ml) or not and examined by immunoblotting with phosphotyrosine-specific monoclonal antibodies PY20-HRP conjugate. For loading controls, FcɛRI β chain-specific antibody was used. Representative immunoblots from three obtained are shown. **(B)** Densitometry analysis of the immunoblots as in **(A)**, in which combined signals from tyrosine-phosphorylated FcεRI β and γ chains in activated cells were normalized to the signals from nonactivated cells and a loading control protein, FcεRI β subunit. **(C)** IgE-sensitized CSK-KD/PAG-KO BMMCs or controls were activated or not with antigen and whole-cell lysates were analyzed by immunoblotting for tyrosine-phosphorylated SYK (pSYK^Y525/Y526^) and LAT (pLAT^Y191^). Tyrosine phosphorylated NTAL (pNTAL) was examined with anti-LAT^Y191^ antibody, which recognizes a common tyrosine-phosphorylated epitope in both LAT and NTAL. For loading controls, SYK- and LAT-specific antibodies were used. Representative immunoblots from 4–6 experiments are shown. **(D–F)** Densitometry analyses of the pSYK^Y525/Y526^ **(D)**, pNTAL **(E)**, and pLAT^Y191^ **(F)** was performed from immunoblotts as in panel **(C)**, in which signals from tyrosine-phosphorylated proteins in activated cells were normalized to the signals from nonactivated cells and corresponding loading control proteins. The results in **(B,D–F)** represent means ± SEM calculated from 3–6 independent experiments. Statistical significance of differences between CSK-KD/PAG-KO and pLKO.1/PAG-KO cells was determined using unpaired two-tailed Student’s *t*-test. *P < 0.05 and **P < 0.01.

To further explore whether PAG has a role at early stages of FcεRI signaling in BMMCs with CSK-KD, we analyzed tyrosine phosphorylation in antigen-activated BMMCs with CSK-KD/PAG-KO and control pLKO.1/PAG-KO cells. The cells were sensitized with IgE, activated or not with antigen for various time intervals, and tyrosine phosphorylation of FcɛRI in the immunoprecipitates was examined (Figure 7A). Quantification of the data showed that tyrosine phosphorylation of FcɛRI β and γ chains was significantly increased in BMMCs with CSK-KD/PAG-KO in comparison with control cells (Figure 7B). We also examined tyrosine phosphorylation of SYK (pSYK^Y525/Y526^), LAT (pLAT^Y191^), and NTAL (pNTAL) in IgE-sensitized and antigen-activated or nonactivated CSK-KD/PAG-KO BMMCs and control pLKO.1/PAG-KO cells (Figure 7C). Statistical evaluation of intergroup differences showed increased tyrosine phosphorylation of SYK^Y525/Y526^ in CSK-KD/PAG-KO cells after antigen stimulation in comparison with control cells (Figures 7C,D). Phosphorylation of LAT^Y191^ and NTAL was also significantly higher in antigen-activated CSK-KD/PAG-KO cells than in control cells (Figures 7C,E,F). Collectively, these data suggest that CSK deficiency is dominant over PAG deficiency in BMMCs with respect to antigen-induced degranulation, Ca^2+^ mobilization, and migration toward antigen and SCF.

## Discussion

**Figure 8:**
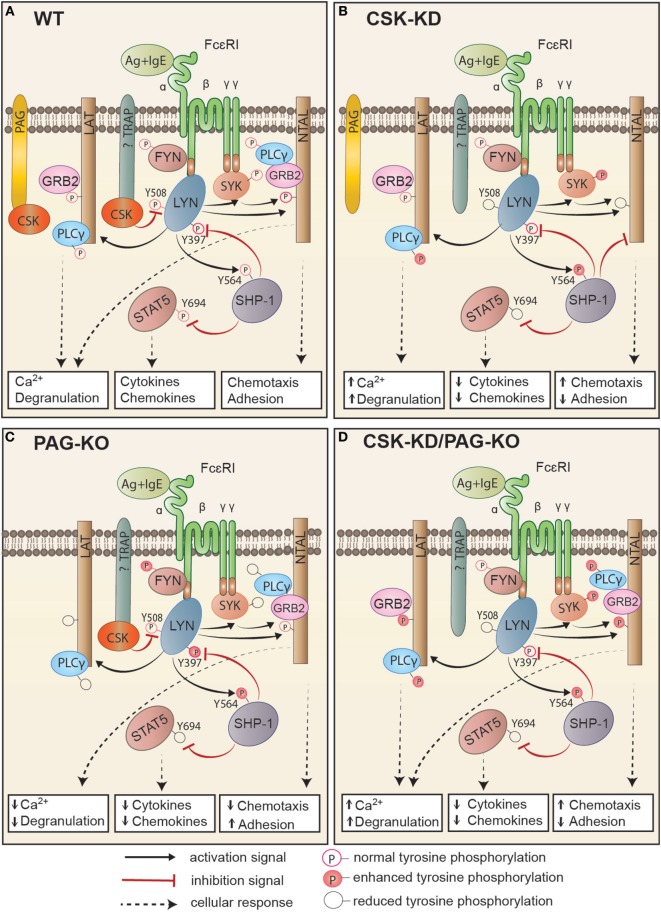
Proposed model of FcεRI signaling in bone marrow-derived mast cells with CSK-KD, PAG-KO, or CSK-KD/PAG-KO. For simplicity, only one FcɛRI with the bound antigen (Ag)–IgE complex is shown. Antigen-activated WT cells **(A)** with normal signal-transduction machinery and cell responses (Ca^2+^ response, degranulation, cytokine and chemokine production, chemotaxis, and adhesion to extracellular matrix proteins) are compared to activated cells with CSK-KD **(B)** or cells with PAG-KO **(C)**. In **(D)**, cells with CSK-KD/PAG-KO are compared with PAG-KO cells **(C)**. Formation of multivalent Ag–IgE–FcɛRI complexes leads to FcɛRI aggregation and tyrosine phosphorylation of FcεRI β and γ subunits by the LYN kinase, followed by binding and activation of SYK and FYN kinases. **(A)** In activated WT cells, CSK binds to phosphorylated PAG and/or other so far not identified transmembrane adaptor protein(s) and phosphorylates the C-terminal inhibitory tyrosine residues of LYN and other Src family tyrosine kinases. Together with PTPs, such as SHP-1, and in cooperation with adaptor proteins, LAT and NTAL, CSK sets a threshold for mast cell activation, which involves GRB2, PLCγ, STAT5, and numerous other molecules that are activated and/or inhibited by phosphorylation/dephosphorylation of various regulatory tyrosines. **(B)** In cells with CSK-KD, phosphorylation of FcɛRI subunits, SYK, and PLCγ is significantly increased, resulting in enhanced calcium response and degranulation. LYN kinase, which is not phosphorylated at the C-terminal inhibitory tyrosine (LYN^Y508^), phosphorylates more efficiently its substrate, SHP-1, at Y^564^, thereby increasing its phosphatase activity, which in turn leads to reduced phosphorylation of STAT5^Y694^. This leads to reduced production of cytokines and chemokines. SHP-1 could also bind to NTAL, but not LAT, and in this way alter the balance between the signaling proteins anchored to these adaptors. Changes in NTAL tyrosine phosphorylation lead to enhanced chemotaxis and reduced adhesion to extracellular matrix proteins. **(C)** Cells with PAG-KO exhibit different changes in the signaling events when compared to WT cells and cells with CSK-KD. PAG KO cells exhibit reduced tyrosine phosphorylation of FcɛRI β and γ subunits, SYK, LAT, PLCγ, and STAT5, resulting in reduced Ca^2+^ response, degranulation, cytokine, and chemokine production, and reduced chemotaxis toward antigen, but enhanced adhesion to fibronectin [Figure 7 of this study and our previous study, Ref. (24)]. **(D)** In cells with CSK-KD/PAG-KO, phosphorylation of SYK, NTAL, LAT, and PLCγ is increased, resulting in enhanced calcium response, degranulation, adhesion, and chemotaxis, but strongly reduced production of TNF-α. Immunoreceptor signaling in cell with CSK-KD/PAG-KO resembles more to signaling of cells with CSK-KD than cells with PAG-KO.

In this study we explored the role of CSK and its plasma membrane anchor PAG in mast cell signaling by examining the activation of BMMCs with CSK-KD or CSK-OE alone or in combination with PAG-KO. Several lines of evidence presented in this study, summarized in Figure 8, indicate that CSK in BMMCs acts as a negative regulator of FcεRI-mediated degranulation, calcium response, and chemotaxis, but as a positive regulator of adhesion to fibronectin and proinflammatory cytokine and chemokine production.

When BMMCs with CSK-KD were activated by antigen-IgE-mediated FcɛRI aggregation, degranulation was significantly increased not only after antigen-induced stimulation but also in resting cells with CSK-KD. This finding, together with enhanced phosphorylation of FcεRI β and γ subunits in nonactivated cells, indicates that CSK contributes to setting the activation threshold in mast cells. This conclusion is consistent with the previous finding that CSK-deficient granulocytes exhibit elevated spontaneous and ligand-induced degranulation (18). Overexpression of CSK in mast cells resulted in reduced mast cell degranulation upon antigen stimulation, which is in accord with the previous observation in rat basophilic leukemia cells (RBL-2H3), in which enhanced expression of CSK delayed histamine release observable in a short period of time upon antigen stimulation (50). Antigen-induced FcεRI aggregation is followed by receptor internalization in which cytoskeletal components play an important role (51–53). Reduced internalization of the FcεRI aggregates is known to prolong generation of the FcεRI-induced signal and, therefore, enhance degranulation (54, 55). Thus, CSK could contribute to enhanced degranulation through its negative regulation of FcεRI internalization.

The calcium response in antigen-stimulated BMMCs was negatively regulated by CSK. This process is initiated by the LYN-SYK-LAT-PLCγ-dependent signaling pathway or complementary FYN-dependent pathway (44). Our data show that CSK-KD results in significantly increased tyrosine phosphorylation of SYK^Y525/Y526^ and PLC-γ^Y783^, but tyrosine phosphorylation of LAT^Y191^ is comparable with control cells. In contrast, tyrosine phosphorylation of NTAL was significantly decreased in antigen-activated BMMCs with CSK-KD. These data are in accord with those showing that overexpression of CSK in a T cell line resulted in significant inhibition of TCR-induced activation events (56) and that in TCR-activated T cells with CSK catalytic activity inhibited by a small inhibitor, phosphorylation of ZAP-70, LAT, and PLCγ was significantly increased when compared to control cells (40).

Mast cell chemotaxis is a complex process involving numerous molecules (39), and it is not clear at which step CSK is involved. One possibility is that CSK affects integrins, which are involved in cell chemotaxis and adhesion. Although we found reduced adhesion to fibronectin in both antigen- and SCF-activated cells, and consistently enhanced migration toward antigen and SCF in cells with CSK-KD, the unchanged presence of β1-integrin on the cell surface suggests different molecular mechanisms involved in these processes. In our previous study we found an important role for NTAL in chemotaxis toward antigen (57). In antigen-activated cells, NTAL was rapidly phosphorylated and served as a negative regulator of degranulation and calcium response (41, 58), a positive regulator of adhesion to fibronectin, and a negative regulator of chemotaxis toward antigen (57), properties that are shared with CSK. NTAL seems to signal to the mast cell cytoskeleton *via* small GTPase RhoA, and it is possible that this pathway is affected by CSK. In fact, it has been already reported that there is a cross-talk between CSK and Rho-family GTPases, which could regulate cell migration events (59).

Our experiments focused on transcription and production of proinflammatory cytokines and chemokines, indicated that CSK is a positive regulator of these processes. These findings are in line with a study using RAW 264.7 macrophage cell line, where CSK was described as a positive regulator of TNF-α and IL-6 production (60). A positive regulatory role of CSK in these processes could be mediated through phosphorylation of the transcription factor STAT5, which is required for transcription of numerous genes involved in various inflammatory processes (61), mast cell development and survival (62), myeloid cell proliferation and differentiation (63), and hematologic malignancies (64). Our finding of reduced tyrosine phosphorylation of STAT5^Y694^ in antigen-activated BMMCs with CSK-KD could be explained by enhanced activity of protein phosphatase SHP-1, which is known to dephosphorylate STAT5^Y694^ (48). This possibility is supported by our observation that SHP-1 co-immunoprecipitates with STAT5. Furthermore, it has been previously found that SHP-1-STAT5 interact in *me^v^/me^v^* BMMCs expressing SHP-1 (48, 49). Previous study also showed that reduced phosphorylation of phosphatase SHP-1^Y536 and^ ^Y564^ resulted in reduced phosphatase activity and constitutive activation of STAT5 (49).

C-terminal Src kinase phosphorylates C-terminal tyrosines of SFKs and in this way inhibits their kinase activity. As expected, phosphorylation of Y^508^ located at the C-terminus of LYN was reduced in BMMCs with CSK-KD when compared with control cells. This is in accord with our previous study (65) indicating importance of this tyrosine for control of both proximal and distal signaling pathways in FcεRI-activated cells. Interestingly, phosphorylation of SFKs at Y^397^ was unchanged and rather slightly decreased in antigen-activated BMMCs with CSK-KD. Although there was a dramatic decrease in phosphorylation of LYN^Y508^ in BMMCs with CSK-KD, we observed rather decrease in phosphorylation of LYN and FYN at Y^397^ accompanied by significantly decreased kinase activity of LYN, but not FYN, in antigen-stimulated BMMCs with CSK-KD. This could be caused in part by co-precipitation of the inhibitory phosphatases. It has been previously described that LYN, but not the other SFKs, possesses a potential SHP-1-recognition site, termed kinase tyrosine-based inhibitory motif, localized mostly within or in the vicinity of the kinase domain (66). This is supported by our data indicating that phosphorylation of SHP-1^Y564^ is strictly dependent on LYN kinase. Highly relevant are data indicating that phosphorylation of SHP-1^Y564^ by LYN kinase is indispensable for maximal phosphatase activity, while phosphorylation of SHP-1^Y536^ is necessary for efficient interaction with STAT5 (49). Additionally, it has been reported that LYN directly interacts with SHP-1 in BMMCs (67). The combined data suggest that CSK modulates SHP-1 phosphorylation through LYN kinase.

To explain the reduced cytokine production in BMMCs with CSK-KD, we propose that LYN that is not phosphorylated at the C-terminal inhibitory tyrosine probably augments SHP-1 activity through SHP-1^Y564^ phosphorylation. Concurrently, SHP-1 could use LYN as a substrate and in this way reduces the LYN kinase activity. Once SHP-1 is activated, it could also dephosphorylate STAT5^Y694^, thereby reducing its activity and leading to inhibition of cytokine/chemokine gene expression. Presence of CSK and STAT5 in SHP-1 immunoprecipitate and co-localization of SHP-1 in Lyn and CSK immunoprecipitates support the existence of such signaling axis. The importance of SHP-1/LYN cross-talk in the regulation of cytokine production was also demonstrated in PLCβ3-deficient BMMCs (67), and relevant are also data indicating that SHP-1-deficient BMMCs exhibited elevated production of proinflammatory cytokines (TNF-α, IL-6, and IL-13), which led to allergic inflammatory responses in the lung tissue (68).

Despite the fact that LAT and NTAL adaptors share structural similarities (3, 69), they are differently phosphorylated, depending on the signaling pathway involved (2, 70). When BMMCs with CSK-KD were compared with pLKO.1 control cells, there was no difference in basal tyrosine phosphorylation of LAT and NTAL. However, after activation, LAT was phosphorylated to the same extent in both cell types, whereas NTAL showed significantly reduced phosphorylation in cells with CSK-KD. The observed difference in phosphorylation of LAT and NTAL in antigen-activated cells with or without CSK could be in part explained by the finding that LAT and NTAL could bind not only positive regulators but also negative regulators, and that there is a competition between LAT and NTAL for binding of the phosphatases (71). It should also be noted that upon FcεRI triggering, SHP-1 binds NTAL, and that mast cells deficient in SHP-1 exhibit significantly increased phosphorylation of this adaptor protein (68). Thus, the reduced phosphorylation of NTAL in BMMCs with CSK-KD could be explained by enhanced activity of SHP-1, which targets NTAL.

C-terminal Src kinase is a cytoplasmic protein that requires binding to adaptor proteins for its localization to the close proximity of plasma membrane signalosomes. A well-known membrane-bound anchor of CSK is PAG, which is involved in the negative regulation of enzymatic activity of SFKs in various cell types (19). Interestingly, our previous report on the biochemical activity and responses of PAG-deficient mast cells (24) provided evidence that in mast cells, PAG functions as a positive regulator of FcɛRI-mediated signaling pathways, and suggested that PAG and CSK have some non-overlapping regulatory functions in the mast cell activation events. In this study, we found that BMMCs with CSK-KD/PAG-KO exhibited enhanced antigen-induced degranulation when compared to pLKO.1 controls in both PAG-KO and WT cells. These data indicate that PAG is not required for CSK functioning in this signaling pathway and that silencing of CSK in PAG-KO BMMCs rescues mast cell degranulation to the cells with CSK-KD. The reduced degranulation in BMMCs with PAG-KO cells and enhanced degranulation in cells with CSK-KD and CSK-KD/PAG-KO suggest that besides PAG, there are other CBPs in mast cells, and that among them there is a competition for CSK. Thus, in the absence of PAG, CSK could bind more efficiently to other adaptors and inhibit relevant SFKs more efficiently. Our previous findings that in mast cells only a relatively small fraction of CSK (~4%) is found in detergent-resistant membranes, that this fraction is not changed after FcεRI triggering, and that this fraction disappears in cell extracts from PAG-KO BMMCs (24) suggest that, in contrast to PAG, the hypothetical CSK anchors reside in the detergent-soluble fraction of the cell extracts.

In our previous study we also found that in PAG-KO cells, the LAT adaptor was less phosphorylated than in control cells (24). In this study, we therefore examined phosphorylation of LAT in cells with CSK-KD/PAG-KO. Our data clearly showed higher LAT phosphorylation in CSK-KD/PAG-KO cells than in PAG-KO cells. Unexpectedly, CSK-KD alone reduced NTAL adaptor protein phosphorylation, but CSK-KD/PAG-KO enhanced its phosphorylation significantly. These data support the notion that CSK and PAG are involved in different signaling pathways and that competition between PAG and hypothetical, not yet identified, CSK anchor plays an important role. A seeming dichotomy was also observed when the role PAG and CSK on mast cell chemotaxis was examined. Chemotaxis toward antigen was positively regulated by PAG (24) but negatively by CSK (this study). In the cells with CSK-KD/PAG-KO, the positive regulatory role of PAG in chemotaxis toward antigen was neutralized by CSK-KD. On the other hand, our finding that CSK is a negative regulator of both antigen- and SCF-mediated chemotaxis, supports the concept that CSK and PAG have different regulatory roles even in these processes. In contrast to other signaling events analyzed, both CSK and PAG exhibited positive regulatory roles in FcεRI-mediated production of proinflammatory cytokine. In the case of TNF-α, the effects of CSK and PAG were additive.

Taken together, our results indicate that CSK in mast cells is a negative regulator of antigen-induced calcium response, degranulation, and chemotaxis toward antigen and SCF, but a positive regulator of cytokine and chemokine production and adhesion to fibronectin. Based on our data we propose that CSK regulates the activity of SHP-1 through the reduced activity of LYN kinase and through this signal circuit affects phosphorylation of STAT5, and proinflammatory cytokines and chemokines. Interestingly, some of the regulatory functions of CSK are not dependent on its binding to PAG, which seems to have other not yet fully understood roles in FcεRI signaling. The opposite regulatory roles of CSK and PAG in antigen-induced degranulation, calcium response, adhesion, and chemotaxis suggest that CSK binds not only to PAG, but also to some other anchors, which could serve for more efficient positioning of CSK in the vicinity of SFKs and thus mediate more potent inactivation of SFKs involved in FcεRI signaling. These alternative CSK anchoring proteins remain to be identified.

## Ethics Statement

This study was carried out in accordance with the recommendation and approval of the Animal Care and Usage Committee of the Institute of Molecular Genetics (Permit number 12135/2010-17210) and national guidelines (law 409/2008), and was in compliance with the EU Directive 2010/63/EU for animal experiments.

## Author Contributions

LP and PD designed the study and wrote the manuscript. LP performed most of the experiments. LD performed chemotactic and kinase assays, immunoprecipitations, and immunoblotting experiments. IH performed flow cytometry analysis of β1-integrin, FcεRI internalization, and RT-qPCR analyses of cytokines and chemokines. TP performed calcium response analysis and immunoblotting experiments. All authors analyzed the data and read and approved the final version of the manuscript.

## Conflict of Interest Statement

The authors declare that the research was conducted in the absence of any commercial or financial relationships that could be construed as a potential conflict of interest.

## Supplementary Material

Figure S1 Production and initial characterization of bone marrow-derived mast cells (BMMCs) with reduced or enhanced expression of CSK. **(A)** Determination of CSK protein by immunoblotting in whole-cell lysates from BMMCs transduced with individual CSK-specific shRNAs (35, 36, 64, or 90) or control empty vector (pLKO.1). **(B)** Quantification of the relative amounts of CSK normalized to the relative amounts of GRB2 used as a loading control and the amount of CSK in pLKO.1 control cells. **(C)** Determination of CSK in whole-cell lysates from BMMCs transduced with a pool of CSK-specific shRNAs (labeled as CSK-KD) or pLKO.1 control vector. **(D)** Quantification of the relative amounts of CSK analyzed as in Figure 1C. **(E)** Determination of CSK by immunoblotting in whole-cell lysates from BMMCs transduced with empty vector (pCDH) or pCDH vector containing the hCSK-mCherry construct (CSK-OE). Positions of endogenous (En.) and exogenous (Ex.) CSKs are indicated by arrows. **(F)** Quantification of the relative amounts of CSK and CSK construct normalized to the relative amounts of GRB2 used as a loading control and the amount of CSK in cells transduced with pCDH control vector. **(G)** Flow cytometry analysis of the surface presence of FcɛRI and c-KIT in BMMCs with CSK-KD, CSK-OE, and appropriate control cells (pLKO.1 and pCDH). Cells not exposed to anti-FcεRI and anti-cKit were also analyzed (non-labeled). **(H)** Quantification of surface FcɛRI and c-KIT, obtained in the experiments as in Figure 1G; fluorescence was normalized to pLKO.1 and pCDH controls. The results in **(B,D,F,H)** represent means ± SEM from 5–13 independent experiments.

Figure S2 Phosphorylation of LYN and FYN at Y397 is unchanged in bone marrow-derived mast cells (BMMCs) with CSK-KD. **(A)** IgE-sensitized BMMCs with CSK-KD or control pLKO.1 cells were activated or not with antigen (250 ng/ml) for 3 min. The cells were lysed and Lyn was immunoprecipitated with LYN-specific antibody. Phosphorylation was analyzed by immunoblotting (IB) with phospho-SFK antibody (pSFK^Y397^). Amount of LYN was determined with Lyn-specific antibody. **(B)** Densitometry analyses of the pSFK^Y397^ were performed from immunoblots as in panel **(A)**, in which signals from tyrosine-phosphorylated proteins in activated cells were normalized to the signals in nonactivated cells and amount of LYN. **(C)** BMMCs were activated as in panel **(A)** and FYN from the cell lysates were immunoprecipitated with FYN-specific antibody. Immunoprecipitates were analyzed by immunoblotting with antibody specific for pSFK^Y397^ and FYN antibody as in panel **(A)**. **(D)** Densitometry analyses of the pSFK^Y397^ were performed from immunoblots as in panel **(C)**, in which signals from tyrosine-phosphorylated FYN proteins in activated cells were normalized to the signals from nonactivated cells and amount of FYN. In **(A,C)** representative immunoblots from three experiments are shown. Means ± SEM were calculated from three independent experiments. Differences between pLKO.1 and CSK-KD in **(B,D)** were not statistically significant as determined using unpaired two-tailed Student’s *t*-test.

Figure S3 Production of PAG-KO bone marrow-derived mast cells (BMMCs) with reduced CSK expression. **(A)** CSK protein expression determined by immunoblotting with whole-cell lysates from PAG-KO BMMCs transduced with CSK-specific shRNAs (pooled shRNAs; CSK-KD) or empty pLKO.1 vector. GRB2 was used as a loading control. **(B)** Quantification of the relative amounts of CSK normalized to the amounts of GRB2 used as a loading control and the CSK level in pLKO.1 control cells. **(C)** Flow cytometry analysis of the surface presence of FcɛRI and c-KIT receptors in PAG-KO BMMCs with CSK-KD and corresponding controls. **(D)** Quantification of surface FcɛRI and c-KIT, obtained in the experiments as in panel **(C)**; fluorescence was normalized to pLKO.1 controls. The data in **(B,D)** represent means ± SEM from 5–8 independent experiments.

## Abbreviations

APC, allophycocyanin; Au-NP, colloidal gold nanoparticles; BMMC, bone marrow-derived mast cells; BSA, bovine serum albumin; CSK, C-terminal Src kinase; FcɛRI, high-affinity IgE receptor; FCS, fetal calf serum; FITC, fluorescein isothiocyanate; HRP, horseradish peroxidase; IL, interleukin; KD, knockdown; KO, knockout; LAT, linker for activation of T cells; mAB, monoclonal antibody; MEM, minimum essential medium; NTAL, non T cell activation linker; NTG, non-target; PAG, phosphoprotein associated with glycosphingolipid-enriched microdomains; PBS, phosphate-buffered saline; OE, overexpressor; PLC, phospholipase C; PTK, protein tyrosine kinase; PTP, protein tyrosine phosphatase; RT-qPCR, real-time quantitative polymerase chain reaction; SCF, stem cell factor; SDS, sodium dodecyl sulfate; SFK, Src family kinase; Sh, short hairpin; SH2, Src homology 2; TBS, tris-buffered saline; TBST, TBS with 0.05% Tween 20; TCR, T cell receptor; TNF, tumor necrosis factor; TNP, 2,4,6-trinitrophenol.
