# Supplementary figures and images for: Positive and Negative Regulatory Roles of C-Terminal Src Kinase (CSK) in FcεRI-Mediated Mast Cell Activation, Independent of the Transmembrane Adaptor PAG/CSK-Binding Protein
Source: Front Immunol. 2018 Aug 2;9:1771. doi: 10.3389/fimmu.2018.01771 (PMC6082945; doi:10.3389/fimmu.2018.01771)

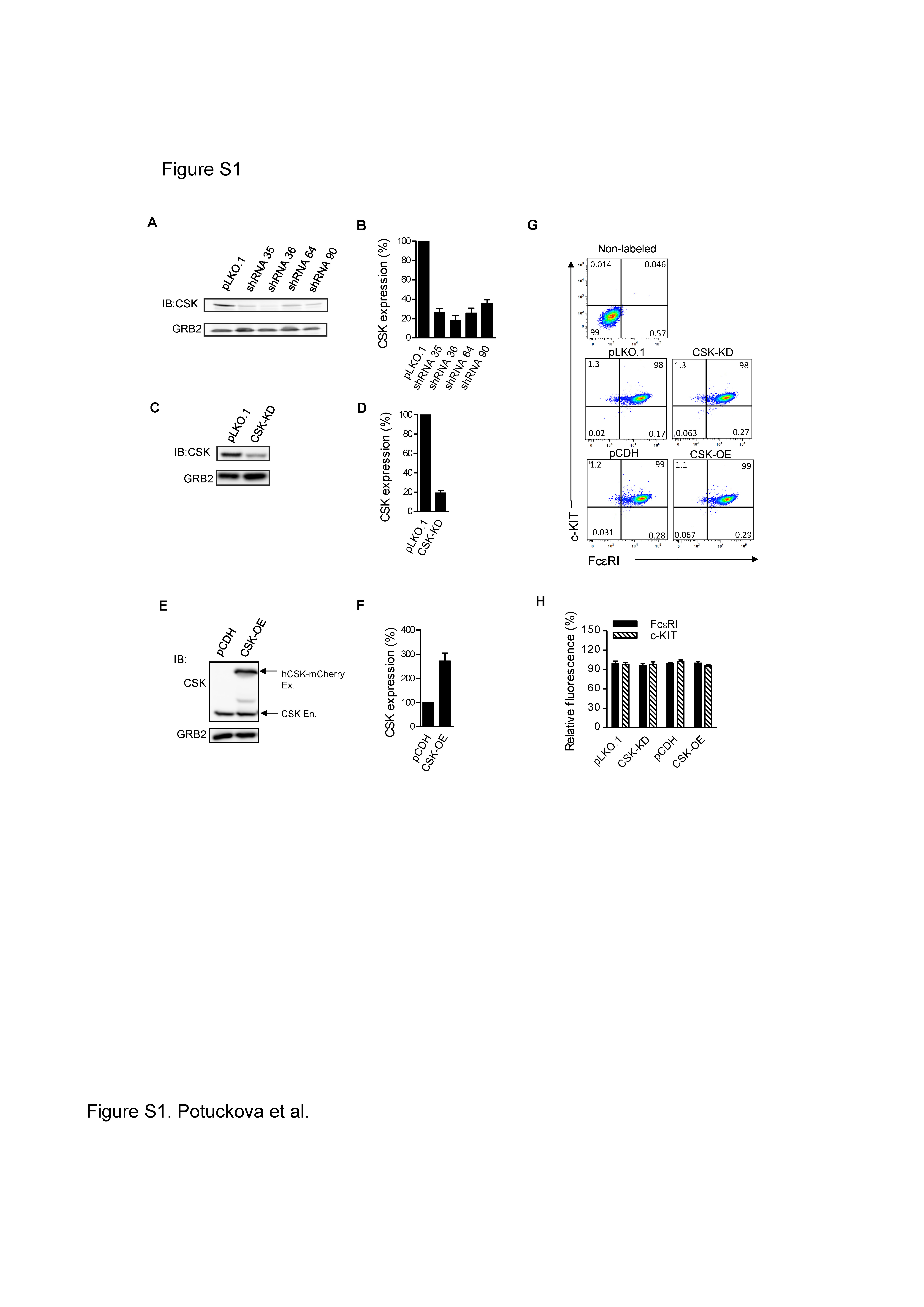

Supplement: Figure S1 — Production and initial characterization of bone marrow-derived mast cells (BMMCs) with reduced or enhanced expression of CSK. (A) Determination of CSK protein by immunoblotting in whole-cell lysates from BMMCs transduced with individual CSK-specific shRNAs (35, 36, 64, or 90) or control empty vector (pLKO.1). (B) Quantification of the relative amounts of CSK normalized to the relative amounts of GRB2 used as a loading control and the amount of CSK in pLKO.1 control cells. (C) Determination of CSK in whole-cell lysates from BMMCs transduced with a pool of CSK-specific shRNAs (labeled as CSK-KD) or pLKO.1 control vector. (D) Quantification of the relative amounts of CSK analyzed as in Figure 1C. (E) Determination of CSK by immunoblotting in whole-cell lysates from BMMCs transduced with empty vector (pCDH) or pCDH vector containing the hCSK-mCherry construct (CSK-OE). Positions of endogenous (En.) and exogenous (Ex.) CSKs are indicated by arrows. (F) Quantification of the relative amounts of CSK and CSK construct normalized to the relative amounts of GRB2 used as a loading control and the amount of CSK in cells transduced with pCDH control vector. (G) Flow cytometry analysis of the surface presence of FcɛRI and c-KIT in BMMCs with CSK-KD, CSK-OE, and appropriate control cells (pLKO.1 and pCDH). Cells not exposed to anti-FcεRI and anti-cKit were also analyzed (non-labeled). (H) Quantification of surface FcɛRI and c-KIT, obtained in the experiments as in Figure 1G; fluorescence was normalized to pLKO.1 and pCDH controls. The results in (B,D,F,H) represent means ± SEM from 5–13 independent experiments. [file image_1.jpeg]

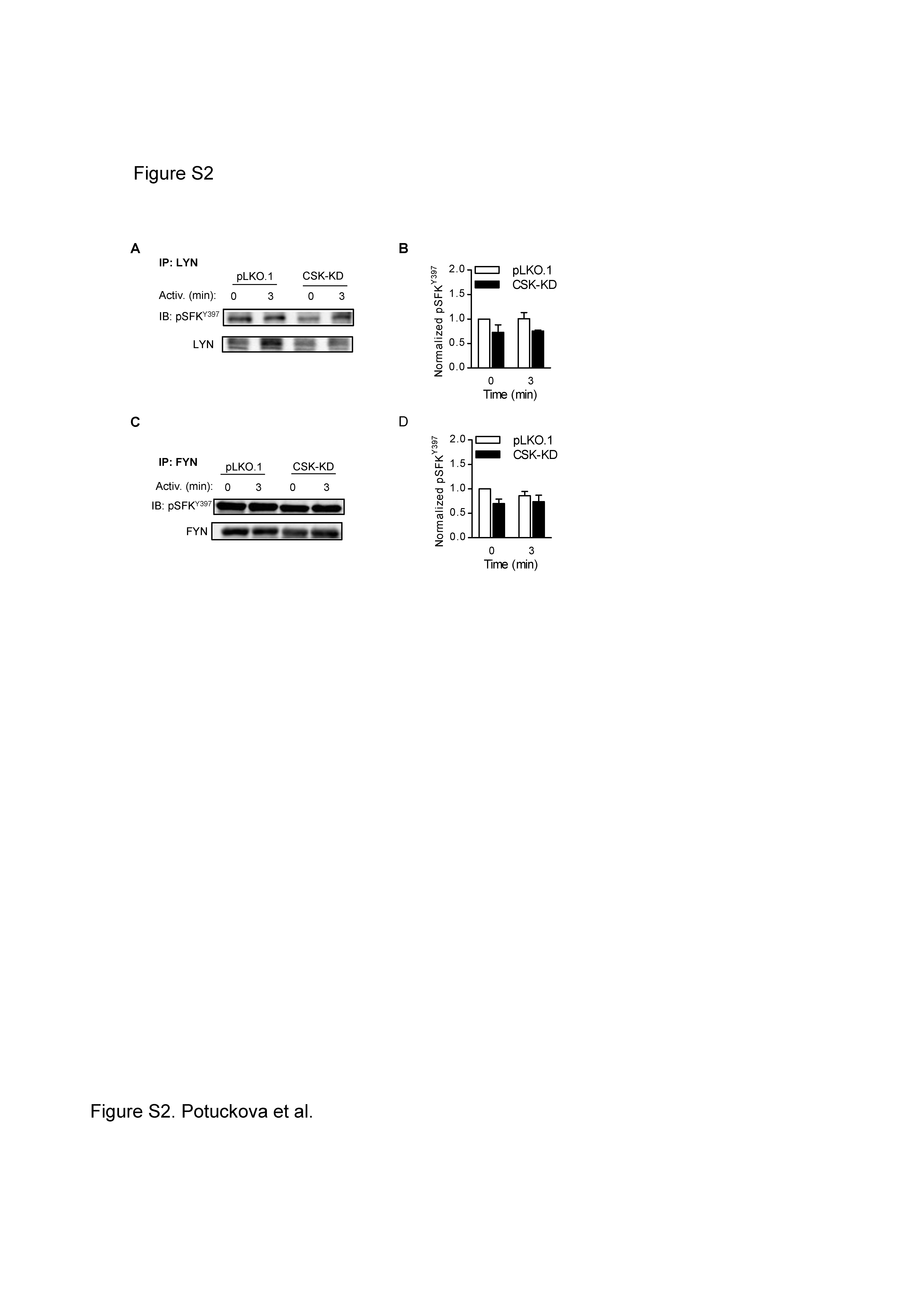

Supplement: Figure S2 — Phosphorylation of LYN and FYN at Y397 is unchanged in bone marrow-derived mast cells (BMMCs) with CSK-KD. (A) IgE-sensitized BMMCs with CSK-KD or control pLKO.1 cells were activated or not with antigen (250 ng/ml) for 3 min. The cells were lysed and Lyn was immunoprecipitated with LYN-specific antibody. Phosphorylation was analyzed by immunoblotting (IB) with phospho-SFK antibody (pSFKY397). Amount of LYN was determined with Lyn-specific antibody. (B) Densitometry analyses of the pSFKY397 were performed from immunoblots as in panel (A), in which signals from tyrosine-phosphorylated proteins in activated cells were normalized to the signals in nonactivated cells and amount of LYN. (C) BMMCs were activated as in panel (A) and FYN from the cell lysates were immunoprecipitated with FYN-specific antibody. Immunoprecipitates were analyzed by immunoblotting with antibody specific for pSFKY397 and FYN antibody as in panel (A). (D) Densitometry analyses of the pSFKY397 were performed from immunoblots as in panel (C), in which signals from tyrosine-phosphorylated FYN proteins in activated cells were normalized to the signals from nonactivated cells and amount of FYN. In (A,C) representative immunoblots from three experiments are shown. Means ± SEM were calculated from three independent experiments. Differences between pLKO.1 and CSK-KD in (B,D) were not statistically significant as determined using unpaired two-tailed Student’s t-test. [file image_2.jpeg]

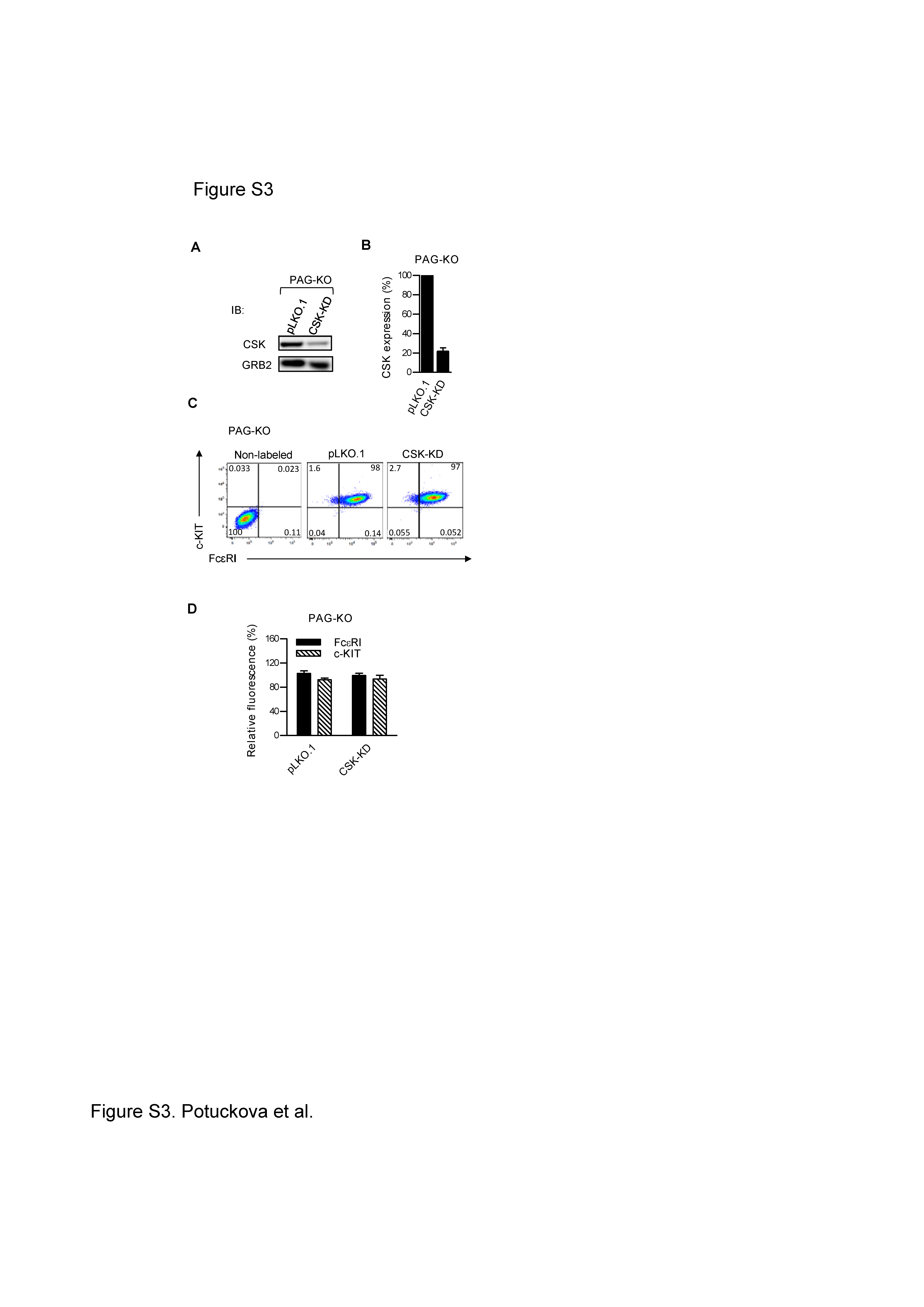

Supplement: Figure S3 — Production of PAG-KO bone marrow-derived mast cells (BMMCs) with reduced CSK expression. (A) CSK protein expression determined by immunoblotting with whole-cell lysates from PAG-KO BMMCs transduced with CSK-specific shRNAs (pooled shRNAs; CSK-KD) or empty pLKO.1 vector. GRB2 was used as a loading control. (B) Quantification of the relative amounts of CSK normalized to the amounts of GRB2 used as a loading control and the CSK level in pLKO.1 control cells. (C) Flow cytometry analysis of the surface presence of FcɛRI and c-KIT receptors in PAG-KO BMMCs with CSK-KD and corresponding controls. (D) Quantification of surface FcɛRI and c-KIT, obtained in the experiments as in panel (C); fluorescence was normalized to pLKO.1 controls. The data in (B,D) represent means ± SEM from 5–8 independent experiments. [file image_3.jpeg]
